# Anti‐Apoptotic and Neurite‐Protective Nanomedicine Augments Embryonic Stem Cells‐Derived Retinal Ganglion Cell Transplantation in Glaucoma Recovery

**DOI:** 10.1002/advs.202513499

**Published:** 2026-02-21

**Authors:** Moxin Chen, Xiaoyan Jiang, Yuanhui Wang, Zhaobin Luo, Yu Zhang, Siwei Liu, Rui Huang, Dandan Zhang, Zhimin Tang, Yahan Ju, Shaohui Pan, Ni Ni, Wei Feng, Yu Chen, Ping Gu

**Affiliations:** ^1^ Department of Ophthalmology Ninth People's Hospital Shanghai Jiao Tong University School of Medicine Shanghai P. R. China; ^2^ Shanghai Key Laboratory of Orbital Diseases and Ocular Oncology Shanghai P. R. China; ^3^ Materdicine Lab School of Life Sciences Shanghai University Shanghai P. R. China; ^4^ School of Medicine Shanghai University Shanghai China; ^5^ State Key Laboratory of Eye Health Eye Hospital Wenzhou Medical University Wenzhou P. R. China

**Keywords:** antioxidative nanomedicine, embryonic stem cells, glaucoma, metal‐polyphenol nanoparticle, retinal ganglion cells

## Abstract

Retinal ganglion cell (RGC) degeneration represents a cardinal etiology of irreversible vision loss in glaucoma, where efficacious regenerative therapies remain scarce. RGC replacement therapy holds promise for visual function restoration, yet its therapeutic efficacy is constrained by the hostile glaucomatous microenvironment, dominated by oxidative stress that compromises transplanted RGC survival. Here, we report the design of multifunctional lithium‐epigallocatechin gallate nanoparticles (Li‐EGCG NPs) to synergistically enhance antioxidant capacity and neuroprotection. These NPs exhibit uniform spherical morphology, broad‐spectrum reactive oxygen species scavenging activity, and exceptional biocompatibility with embryonic stem cell‐derived RGCs (ESC‐RGCs). In vitro, Li‐EGCG NPs mitigate oxidative stress‐induced apoptosis, preserve neurite integrity, and maintain mitochondrial homeostasis in ESC‐RGCs. Transcriptomic analyses reveal activation of neuroprotective pathways, including apoptosis regulation, axon guidance, and mitochondrial function. In a retinal ischemia/reperfusion injury model, which serves as an acute pathological glaucomatous injury model, combinatorial Li‐EGCG NPs and ESC‐RGCs transplantation markedly improves ESC‐RGCs survival, preserves retinal architecture, and restores visual function beyond single‐modality therapy. This study establishes Li‐EGCG NPs as a robust nanomedicine platform for retinal neurodegenerative diseases, offering a promising strategy to augment cell‐based therapies.

## Graphical abstract

Lithium‐epigallocatechin gallate nanoparticles (Li‐EGCG NPs) are co‐transplanted with embryonic stem cell‐derived retinal ganglion cells (ESC‐RGCs) to enhance cell survival and therapeutic efficacy in an acute pathological glaucomatous injury model. This synergistic approach protects RGC cells, preserves retinal structure and improves visual function, highlighting a promising combinatorial nanomedicine strategy for retinal neurodegenerative disease treatment.

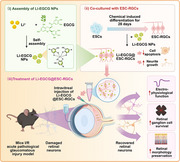

## 1 Introduction

Glaucoma is a progressive neurodegenerative disease and a leading cause of irreversible blindness worldwide, affecting over 80 million people [1]. The disease is characterized by the progressive loss of retinal ganglion cells (RGCs) and their axons, leading to irreversible visual field defects and eventual vision loss. Current clinical interventions, such as intraocular pressure (IOP) lowering medications and surgical procedures, can delay disease progression but fail to reverse the damage once RGCs are lost [2]. Consequently, there remains a critical unmet need for effective therapeutic strategies that can restore visual function by protecting or replacing damaged RGCs. The pathophysiology of glaucoma is multifactorial and complex, involving a combination of oxidative stress, ischemia, and mechanical stress. Among these, oxidative stress‐induced apoptosis and axon degeneration has been identified as a primary contributor to RGC dysfunction [3, 4, 5]. Elevated levels of reactive oxygen species (ROS) disrupt mitochondrial homeostasis, trigger cytochrome c release, and activate caspase‐dependent apoptotic pathways, ultimately leading to RGC death [6, 7]. Furthermore, mitochondrial dysfunction compromises ATP production, which is critical for maintaining axonal transport, synaptic transmission and overall neuronal survival. [8, 9] These processes collectively exacerbate RGC vulnerability and accelerate neurodegeneration, culminating in irreversible vision loss characteristic of glaucoma.

RGC replacement therapy has emerged as a promising approach to restoring visual function by repopulating the retina with new functional RGCs. Among available cell sources, retinal ganglion cells derived from pluripotent stem cells, mainly embryonic stem cells (ESCs) and induced pluripotent stem cells (iPSCs), are considered particularly valuable. Compared with iPSCs, ESCs do not carry epigenetic memory from donor cells and possess a stronger capacity to differentiate into fully mature, functional terminal cell types, thus holding broader application potential [10, 11]. However, despite the theoretical promise, the survival of transplanted RGCs remains severely limited. The glaucomatous microenvironment, dominated by high oxidative stress, poses a hostile niche that hampers cell survival, axon extension, and synaptic connectivity [12, 13]. Notably, generating ESC‐derived RGCs (ESC‐RGCs) involves a carefully controlled, multi‐stage differentiation process requiring at least 28 days, making these cells an extremely precious and scarce resource [14]. Therefore, strategies that can improve the viability and function of transplanted RGCs are urgently needed to advance RGC replacement therapy toward clinical translation.

Epigallocatechin gallate (EGCG), a major polyphenolic component of green tea, has demonstrated potent antioxidant and anti‐apoptotic properties in retinal models [15, 16]. EGCG effectively scavenges ROS, protects retinal neuronal cells from oxidative damage, and preserves visual function in various models of retinal degeneration, making it a promising neuroprotective agent for the retina [17]. In addition, lithium ions (Li^+^), widely used and well‐tolerated in clinical practice, have shown neuroprotective effects through modulation of apoptosis‐related signaling pathways, and promotion of neuronal survival in central nervous system injury and neurodegenerative disease models [18, 19]. In recent years, metal‐polyphenol networks (MPNs) have emerged as a promising platform for biomedical applications due to their facile assembly, excellent biocompatibility, and multifunctionality [20]. By coordinating metal ions with polyphenols, MPNs can simultaneously integrate and potentiate the biological activities of both components, improve stability and enhance antioxidant and cytoprotective effects [20, 21]. Importantly, both EGCG and Li^+^ have demonstrated favorable biosafety profiles, which are crucial for supporting the survival and functional integration of delicate and valuable cell sources such as ESC‐RGCs. Incorporating EGCG and Li^+^ into an MPN structure thus offers a synergistic and biocompatible strategy to protect retinal neurons, modulate the oxidative microenvironment, and enhance the therapeutic potential of cell‐based interventions for glaucoma.

**SCHEME 1:**
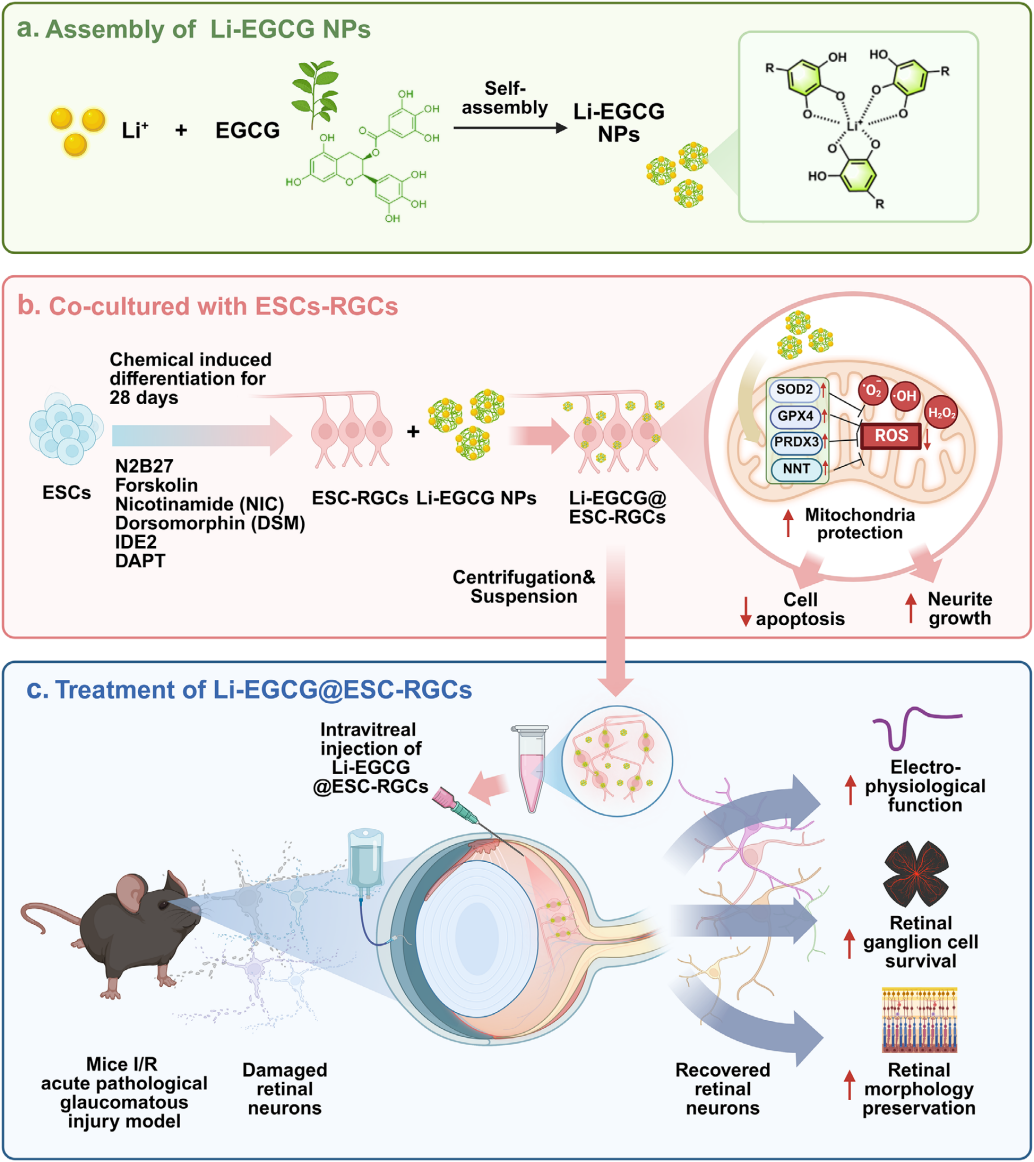
Schematic illustration of Li‐EGCG@ESC‐RGCs for the treatment of glaucoma. (a) Li^+^ and EGCG were mixed and self‐assembled to prepare Li‐EGCG nanoparticles (NPs). (b) Li‐EGCG NPs were applied to treat embryonic stem cell‐derived retinal ganglion cells (ESC‐RGCs), increasing the expression of antioxidant enzymes and protect mitochondria. (c) The retinal ischemia/reperfusion (I/R) acute pathological glaucomatous injury model was established by anterior chamber injection of saline to induce acute intraocular hypertension. Li‐EGCG@ESC‐RGCs were then intravitreally injected into the mouse eye, promoting visual function restoration, RGC survival, and recovery of retinal morphology.

In this study, we designed and synthesized novel lithium‐epigallocatechin gallate nanoparticles (Li‐EGCG NPs) aimed at overcoming the challenges associated with RGC transplantation in glaucoma (Scheme 1). By integrating Li^+^ and EGCG into a stable nanocomplex, we sought to harness their complementary neuroprotective mechanisms to enhance the survival and neurite growth of ESC‐RGCs. We systematically characterized the physicochemical properties and ROS scavenging capacity of Li‐EGCG NPs, assessed their effects on apoptosis, neurite integrity, and mitochondrial homeostasis in vitro, and further validated their therapeutic efficacy in an established retinal ischemia/reperfusion (I/R) acute pathological glaucomatous injury model [22]. Our findings demonstrate that Li‐EGCG NPs not only mitigate the hostile retinal microenvironment but also synergistically promote the survival of transplanted ESC‐RGCs, providing a promising nanomedicine platform for advancing cell‐based therapies in retinal neurodegenerative diseases.

## 2 Results and Discussion

### 2.1 Design, Fabrication, and Characterizations of Li‐EGCG NPs

**FIGURE 1:**
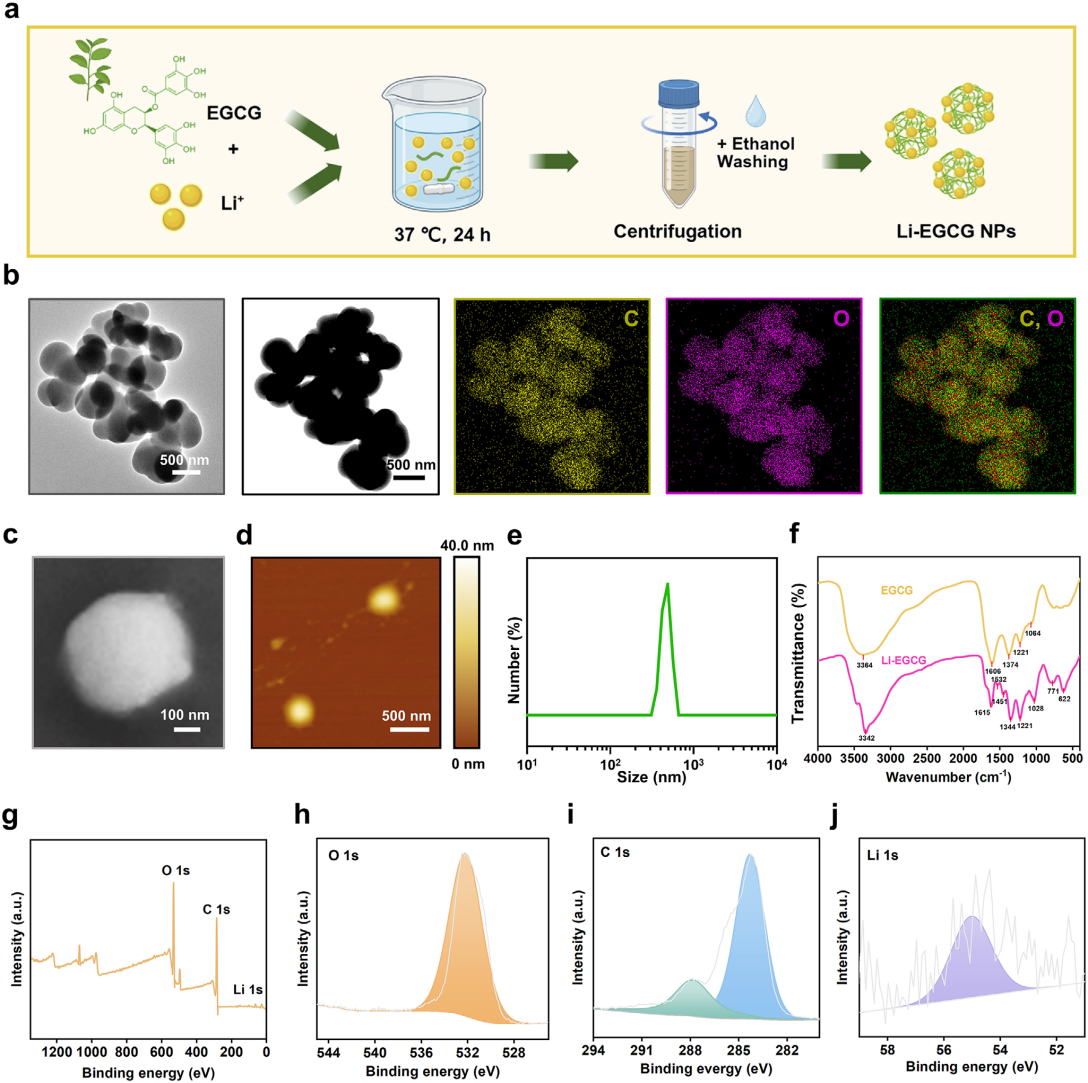
Structure and compositional characterization of Li‐EGCG NPs. (a) Schematic illustration of the preparation of Li‐EGCG NPs. (b) TEM image and elemental mapping of Li‐EGCG NPs. (c) SEM image of Li‐EGCG NPs. (d) AFM image of Li‐EGCG NPs. (e) Size distribution of Li‐EGCG NPs. (f) FTIR spectra of EGCG and Li‐EGCG NPs. (g) XPS spectrum of Li‐EGCG NPs. (h–j) High‐resolution XPS spectra for (h) O 1s, (i) C 1s, and (j) Li 1s.

Li‐EGCG NPs were synthesized via coordination of Li^+^ ions with EGCG under neutral pH conditions, followed by gentle stirring for 24 h, resulting in a clear and transparent solution (Figure 1a). Transmission electron microscopy (TEM) reveals that the resulting nanoparticles possessed a uniform and well‐defined spherical morphology (Figure 1b). Elemental analysis energy‐dispersive X‐ray spectroscopy (EDS) mapping further demonstrates a homogeneous distribution of oxygen (O) and carbon (C) elements throughout the nanoparticles, consistent with the polyphenolic nature of EGCG. However, no Li signal is observed in the EDS spectra, likely due to the low atomic number and light atomic mass of lithium, which limits its detectability in conventional EDS analysis [23, 24]. To further examine the morphology and surface characteristics of the Li‐EGCG NPs, scanning electron microscopy (SEM) and atomic force microscopy (AFM) were performed. The SEM image corroborates the spherical morphology observed in TEM (Figure 1c), while AFM analysis confirms a consistent topography (Figure 1d), reinforcing the uniformity of the nanoparticles. Dynamic light scattering (DLS) measurement reveals that the hydrodynamic diameter of Li‐EGCG NPs is approximately 460 nm (Figure 1e).

To confirm the coordination of Li^+^ in the nanocomplex, Fourier transform infrared (FTIR) spectroscopy reveals that the O–H stretching vibration shifted from 3364 to 3342 cm^−1^, while the C = O stretching band shifts from 1606 to 1615 cm^−1^, compared with free EGCG (Figure 1f), which suggests that the hydroxyl and carbonyl functional groups are involved in Li^+^ coordination, contributing to the self‐assembly and structural stabilization of the Li‐EGCG nanocomplex. Similar vibrational changes have been observed in other EGCG‐based metal coordination systems, such as Sm^III^‐EGCG and Au‐EGCG, indicating a common coordination mechanism involving aromatic hydroxyl and carbonyl groups [25, 26]. To further confirm the elemental composition and successful incorporation of lithium, X‐ray photoelectron spectroscopy (XPS) analysis was performed. The survey spectrum and high‐resolution spectra confirmed the presence of O, C and Li (Figure 1g–j), validating the successful integration of Li^+^ within the nanostructure.

### 2.2 Broad‐Spectrum ROS Scavenging Capability of Li‐EGCG NPs

Oxidative stress is a critical pathological factor in RGC degeneration and significantly limits the survival and integration of transplanted cells [27]. To address this challenge, the broad‐spectrum ROS scavenging capacity of Li‐EGCG NPs was systematically evaluated against representative reactive species, including 2,2’‐azino‐bis (3‐ethylbenzothiazoline‐6‐sulfonic acid) radical ion (ABTS·^+^), 2,2‐diphenyl‐1‐picrylhydrazyl radical (DPPH·), hydroxyl radical (∙OH), superoxide anion radical (·O_2_^−^), hydrogen peroxide (H_2_O_2_), and singlet oxygen (^1^O_2_).

**FIGURE 2:**
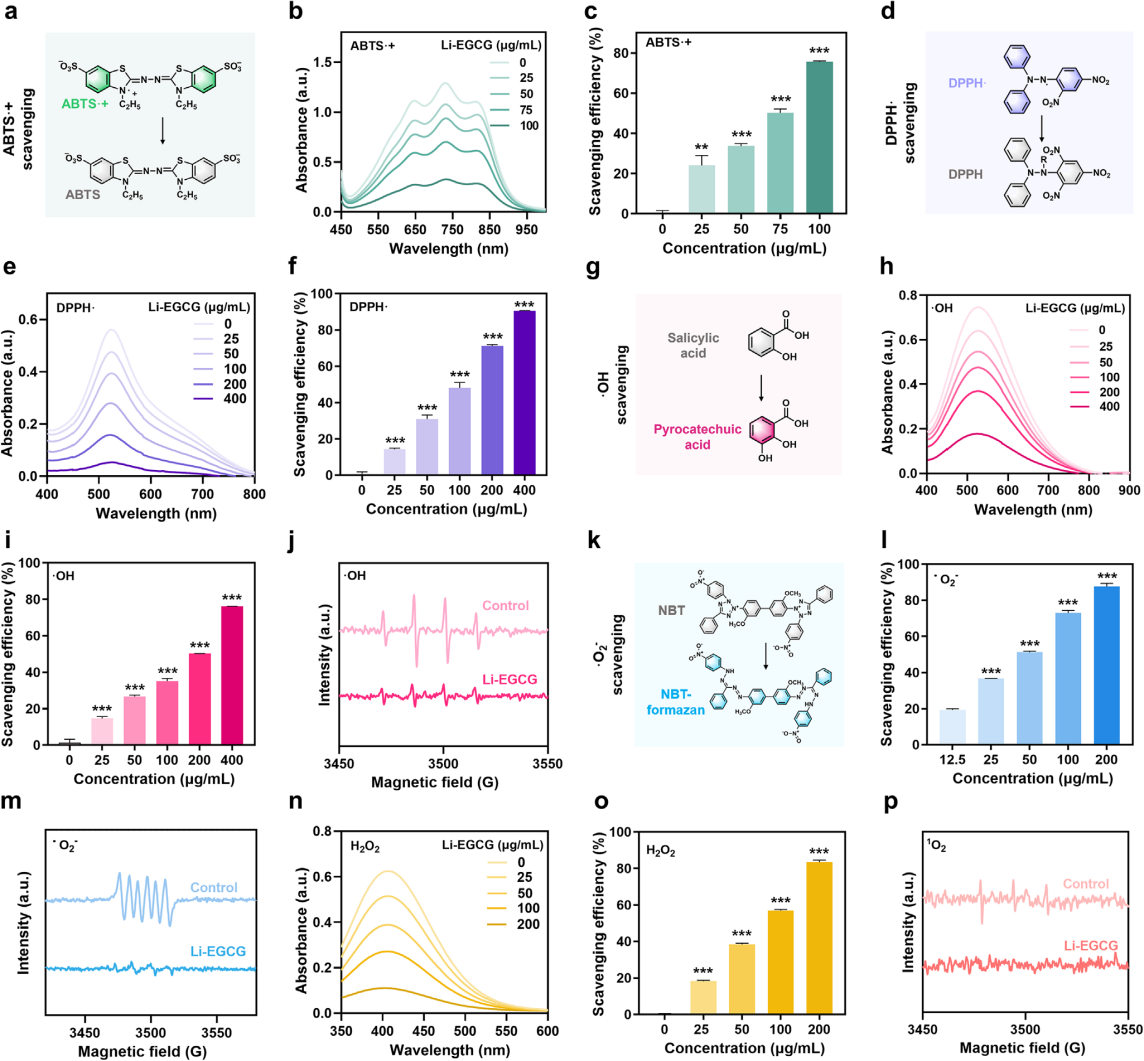
The antioxidant capacity of Li‐EGCG NPs. (a) Schematic depiction of the redox reaction involving ABTS·^+^. (b)Representative UV–vis absorption spectra of the antioxidative performance of Li‐EGCG NPs against ABTS·^+^. (c) ABTS·^+^ scavenging efficiency of Li‐EGCG NPs in a concentration‐dependent manner. (d) Reaction mechanism illustrating the redox process of DPPH·. (e) Representative UV–vis absorption spectra of DPPH· scavenging by Li‐EGCG NPs. (f) Concentration‐dependent DPPH· scavenging activity of Li‐EGCG NPs. (g) Chemical equations representing the redox reactions with ·OH. (h) Representative UV–vis absorption spectra of the antioxidative performance of Li‐EGCG NPs against ·OH. (i) Concentration‐dependent ·OH scavenging efficiency of Li‐EGCG NPs. (j) ESR spectra of ·OH signal trapped by DMPO. (k) Redox reaction pathway of ^·^O_2_^−^ scavenging. (l) Concentration‐dependent ^·^O_2_^−^ scavenging activity of Li‐EGCG NPs. (m) ESR spectra of ^·^O_2_^−^ signal trapped by DMPO. (n) Representative UV–vis absorption spectra of the concentration‐dependent H_2_O_2_ scavenging performance of Li‐EGCG NPs. (o) Concentration‐dependent scavenging efficiencies of H_2_O_2_. (p) ESR spectra of ^1^O_2_ signal trapped by TEMP. Data are presented as mean ± SD; statistical analysis was performed using one‐way ANOVA followed by Tukey's post hoc tests (^*^ *p* < 0.05, ^**^ *p* < 0.01, ^***^ *p* < 0.001).

To begin with, the antioxidant capacity of Li‐EGCG NPs was assessed using the ABTS·^+^ assay (Figure 2a). At a concentration of 100 µg/mL, Li‐EGCG NPs achieve a radical scavenging efficiency of 75.6% toward ABTS·^+^ (Figure 2b,c), indicating a strong ability to neutralize organic radicals. Complementary analysis using the DPPH· assay further confirms this activity, with Li‐EGCG NPs displaying a clear dose‐dependent scavenging effect, reaching 90.6% at 400 µg/mL (Figure 2d–f). The ·OH scavenging activity was evaluated via both the salicylic acid (SA) colorimetric method and electron spin resonance (ESR) spectroscopy. In the SA‐based assay, ·OH oxidizes salicylic acid to generate 2,3‐pyrocatechuic acid, producing a measurable absorbance at 510 nm (Figure 2g). A concentration‐dependent reduction in absorbance indicates effective·OH neutralization by Li‐EGCG NPs (Figure 2h,i). This is corroborated by ESR spectroscopy using DMPO as a spin‐trapping agent, where the characteristic DMPO‐·OH signal is markedly attenuated following nanoparticle treatment (Figure 2j; Figure S1), confirming robust ·OH scavenging. ·O_2_^−^ scavenging ability of Li‐EGCG NPs was evaluated using both the nitroblue tetrazolium (NBT) assay and ESR. In the NBT assay, ·O_2_^−^ reduces NBT to a blue‐colored formazan product, detected at 560 nm (Figure 2k). Treatment with Li‐EGCG NPs results in a concentration‐dependent suppression of formazan formation, indicating ·O_2_^−^ elimination (Figure 2l). ESR analysis shows DMPO‐·O_2_^−^ signal is significantly decreased in the presence of Li‐EGCG NPs (Figure 2m; Figure S2), confirming their potent scavenging effect. H_2_O_2_ scavenging was quantified using the titanium sulfate spectrophotometric (TSS) method, which measures the formation of a yellow peroxytitanium complex absorbing at 410 nm. As the nanoparticles concentration increased, a notable decline in absorbance is observed (Figure 2n,o), suggesting efficient decomposition of H_2_O_2_ by Li‐EGCG NPs. Lastly, the ability of Li‐EGCG NPs to quench ^1^O_2_ was assessed using ESR with 2,2,6,6‐tetramethylpiperidine (TEMP) as a specific spin trap. Upon reaction with ^1^O_2_, TEMP forms 2,2,6,6‐tetramethylpiperidine‐1‐oxyl (TEMPO), a stable nitroxide radical with a characteristic ESR signal. Treatment with Li‐EGCG NPs significantly reduce the TEMPO signal intensity (Figure 2p; Figure S3), indicating effective ^1^O_2_ scavenging. These results demonstrate that Li‐EGCG NPs possess robust and broad‐spectrum antioxidant activity, capable of neutralizing a wide array of ROS.

### 2.3 ESC‐RGCs Differentiation and Biocompatibility With Li‐EGCG NPs

**FIGURE 3:**
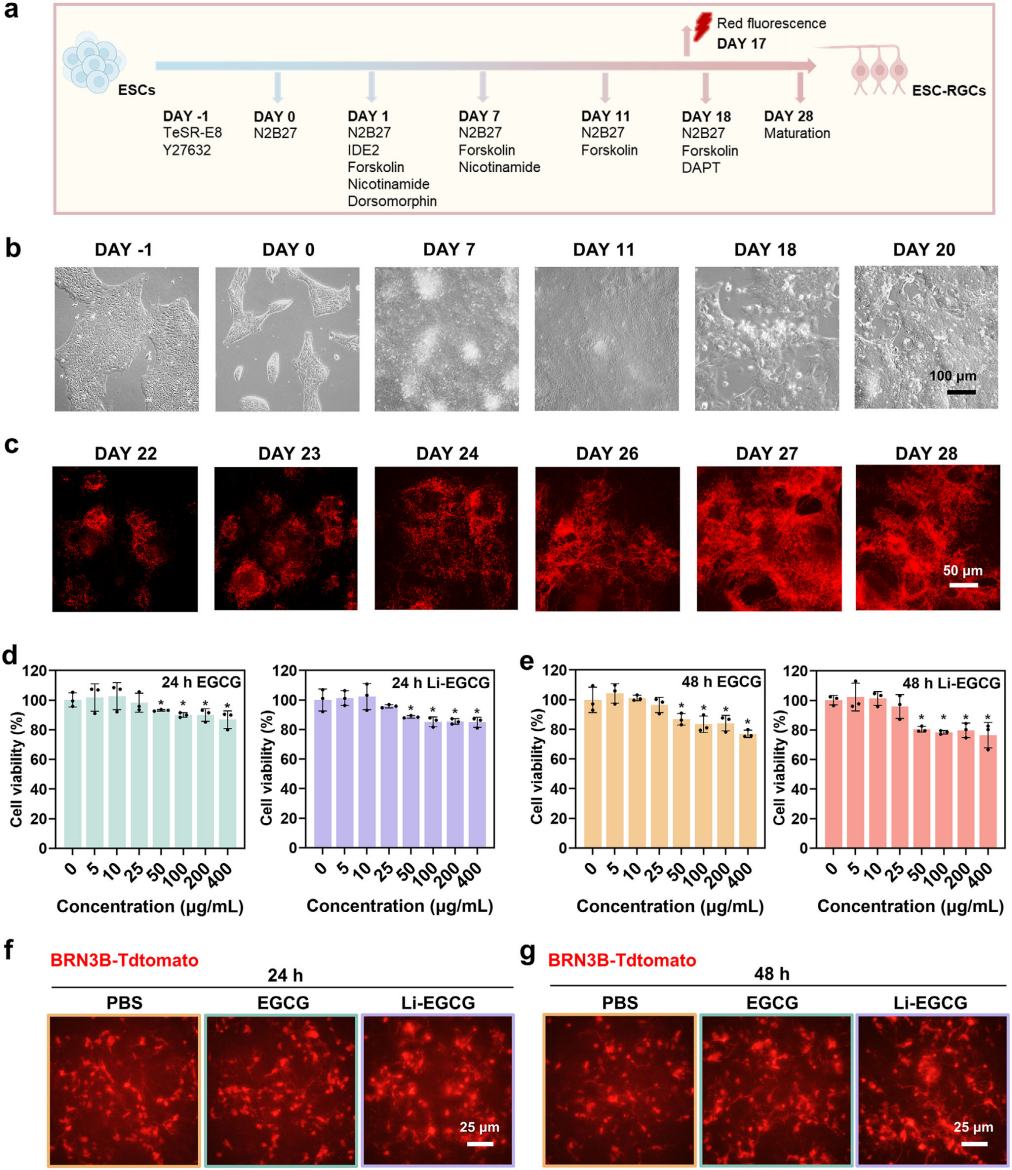
The in vitro biocompatibility assessment of Li‐EGCG NPs. (a) Schematic illustration of the stepwise differentiation protocol used to generate ESC‐RGCs. (b) Representative images showing the morphological progression of ESC‐RGCs from day 1 to day 20 during differentiation. (c) Fluorescence images showing BRN3B‐Tdtomato expression from day 22 to day 28. (d,e) CCK‐8 assay quantifying cell viability of differentiated ESC‐RGCs after d) 24 and (e) 48 h of treatment with varying concentrations of free EGCG (left) or Li‐EGCG NPs (right) (*n* = 3) compared to the untreated group (0 µg/mL). (f,g) Fluorescence images showing robust cell survival and neurite outgrowth in ESC‐RGCs following treatment with 25 µg/mL EGCG or Li‐EGCG NPs for (f) 24 h and (g) 48 h. Data are presented as mean ± SD; statistical analysis was performed using one‐way ANOVA followed by Tukey's post hoc tests (^*^ *p* < 0.05, ^**^ *p* < 0.01 and ^***^ *p* < 0.001).

RGCs are the primary neuronal subtype affected in glaucoma, yet the absence of reliable and renewable RGCs has long hindered mechanistic studies and therapeutic development [27, 28, 29]. To overcome this limitation, we employed a differentiation protocol to generate RGCs from human ESCs. In this study, the well‐characterized H9 human ESCs line, which exhibits typical colony morphology under microscopy, featuring tightly packed cells with well‐defined borders (Figure S4). RGCs differentiation was induced using a fully chemically defined, serum‐free protocol, enabling efficient and reproducible lineage specification (Figure 3a) [14]. To facilitate cell tracking and in vivo monitoring following transplantation, we employed CRISPR/Cas9‐mediated genome editing to knock in a TdTomato fluorescent reporter into the endogenous *BRN3B* locus, a transcription factor selectively expressed in RGCs. This strategy enabled real‐time visualization of differentiating and mature ESC‐RGCs. During differentiation, ESCs progressively transitioned from a pluripotent morphology to form neural rosette‐like structures and subsequently acquired neuronal‐like phenotypes (Figure 3b; Figure S5), indicating successful lineage commitment. By day 17, Tdtomato‐positive cells emerge, and by day 28, mature RGC‐like cells exhibiting robust fluorescence are readily detectable (Figure 3c; Figure S6). To confirm RGC identity, immunocytochemical staining was performed for multiple RGC‐specific markers, including βIII‐tubulin (Tuj1), ISL LIM homeobox 1 (Islet1), and RNA‐binding protein mRNA processing factor (RBPMS) (Figure S7). The co‐expression of these markers with TdTomato fluorescence confirms the successful derivation of authentic RGCs from human ESCs.

To determine a safe and effective working concentration for ESC‐RGCs, a dose‐response analysis was conducted. Cells were treated with increasing concentrations of EGCG or Li‐EGCG NPs, ranging from 0 to 400 µg/mL. Cell viability was measured at 24 and 48 h post‐treatment using the cell counting kit‐8 (CCK‐8) assay (Figure 3d,e). A concentration of 25 µg/mL is identified as a well‐tolerated, with no significant reduction in ESC‐RGC viability. In addition, intracellular ROS levels were evaluated by CellROX staining in ESC‐RGCs treated with Li‐EGCG nanoparticles across the same concentration range. These analyses revealed that 25 µg/mL achieved efficient ROS scavenging (Figures S8 and S9). Accordingly, balancing biosafety and antioxidant efficacy, 25 µg/mL was selected as the working concentration for all subsequent experiments. Fluorescence imaging of ESC‐RGCs expressing TdTomato confirms that treatment with EGCG or Li‐EGCG NPs do not impair cell morphology or neurite outgrowth over 24 and 48 h (Figure 3f,g). To further assess biocompatibility across a broader range of retinal cell types, three additional cell lines, including ARPE‐19 (human retinal pigment epithelial cells), 661 W (mouse photoreceptor cells), and rRMC (rat retinal Müller cells), were tested. Calcein acetoxymethyl ester/propidium iodide (Calcein‐AM/PI) double staining demonstrates a consistently high proportion of viable cells across all treatment groups, with no observable increase in cell death in response to Li‐EGCG NPs (Figure S10), supporting their excellent cytocompatibility.

### 2.4 In Vitro Anti‐Apoptotic and Neurite Protective Effects of Li‐EGCG NPs

**FIGURE 4:**
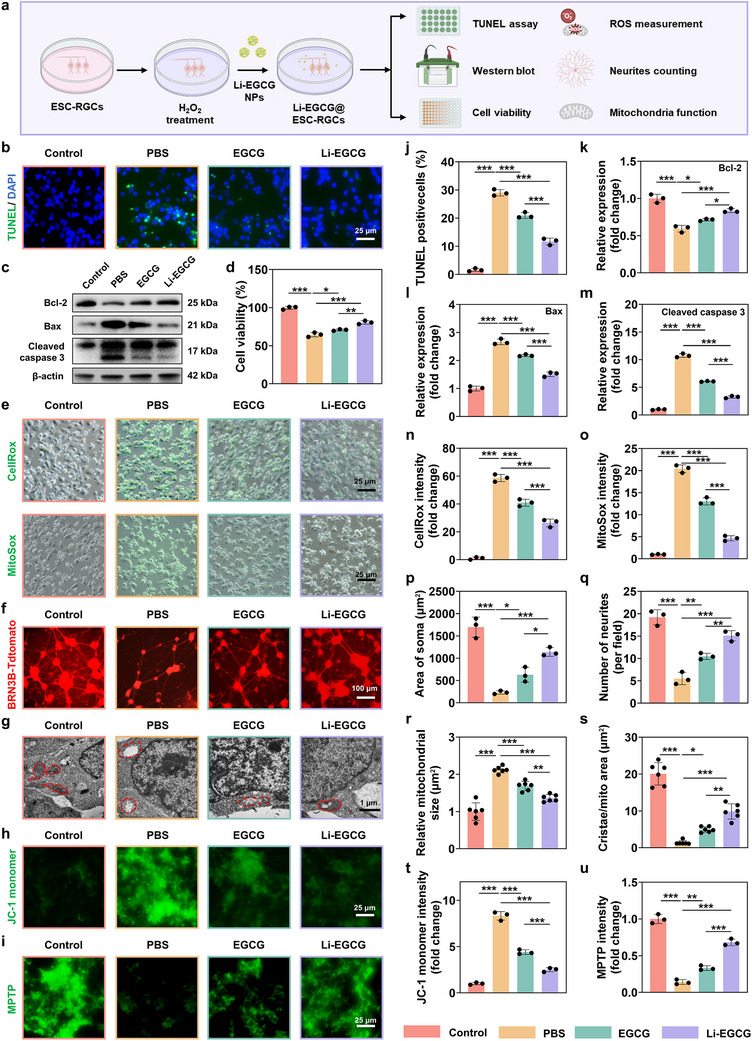
Anti‐apoptotic and neurite‐protective effects of Li‐EGCG NPs. (a) Schematic illustration showing ESC‐RGCs exposed to H_2_O_2_, followed by treatment with Li‐EGCG NPs and subsequent functional assessments. (b) Representative fluorescence images of TUNEL (green) to detect apoptotic cells, and nuclei counterstained with DAPI (blue). (c) Western blot used to determine the expressions of Bcl‐2, Bax, and Cleaved caspase 3. (d) Cell viability evaluated by CCK‐8 assay after H_2_O_2_ treatment (*n* = 3). (e) Representative images of oxidative stress markers: CellRox (green, top row) for overall cellular reactive ROS, and MitoSox (green, bottom row) for mitochondrial ROS. (f) Fluorescence images showing cell survival and neurite morphology in each group after H_2_O_2_ treatment. (g) Bio‐TEM images showing the ultrastructural morphology of ESC‐RGCs, mitochondria are highlighted with red circles. (h) The mitochondrial membrane potential is measured by the JC‐1 monomer (green) fluorescent probe. (i) The MPTP channel measured using the mitochondrial permeability transition pore assay kit. (j) Quantification of TUNEL‐positive cells, presented as the percentage of apoptotic cells in each group (*n* = 3). (k–m) Quantitative analysis of protein expression levels of k) Bcl‐2, l) Bax, and m) Cleaved caspase 3 in each group (*n* = 3). (n,o) Quantitative analysis of ROS levels in each group based on fluorescence intensity of (n) CellROX and (o) MitoSOX staining (*n* = 3). (p,q) Morphometric quantification of (p) soma area and (q) neurite number per cell (*n* = 3). (r,s) Mitochondrial structural quantification based on bio‐TEM images, where (r) average mitochondrial size and (s) cristae number per mitochondrial area (*n* = 6). (t) Quantitative analysis of mitochondrial membrane potential in each group based on fluorescence intensity of JC‐1 monomer staining (*n* = 3). (u) Quantitative analysis of mitochondrial permeability transition pore opening in each group based on the fluorescence intensity of MPTP staining (*n* = 3). A decrease in MPTP staining fluorescence intensity reflects enhanced MPTP opening, whereas higher fluorescence indicates preserved mitochondrial integrity. Red (Control), yellow (PBS), green (EGCG), and purple (Li‐EGCG NPs). Data are presented as mean ± SD; statistical analysis was performed using one‐way ANOVA followed by Tukey's post hoc tests (^*^ *p* < 0.05, ^**^ *p* < 0.01 and ^***^ *p* < 0.001).

To evaluate the neuroprotective efficacy of Li‐EGCG NPs in vitro, oxidative injury was induced in ESC‐RGCs using H_2_O_2_, a well‐established model for simulating oxidative stress‐mediated neurodegeneration [30, 31, 32]. The protective effects were assessed using terminal deoxynucleotidyl transferase dUTP nick end labeling (TUNEL) staining, western blot, CCK‐8 assay, ROS probe staining, neurite analysis, and mitochondria functional analyses (Figure 4a). Both EGCG and Li‐EGCG NPs significantly reduce H_2_O_2_‐induced apoptosis, with Li‐EGCG NPs showing a more pronounced anti‐apoptotic effect (Figure 4b,j), suggests that Li^+^ coordination enhances the cytoprotective capacity of EGCG. To further elucidate the anti‐apoptotic effects, Western blot analysis showed that H_2_O_2_ exposure decreased Bcl‐2 expression while increasing Bax and cleaved caspase‐3 levels. Both EGCG and Li‐EGCG NPs reversed these apoptotic changes, with Li‐EGCG NPs exerting a more pronounced effect (Figure 4c,k,l,m). Li^+^ are known for their neuroprotective and anti‐apoptotic properties in various neuronal models [33, 34, 35]. Mechanistically, Li^+^ can inhibit pro‐apoptotic signaling pathways such as cytochrome c release and caspase activation, while modulating a range of cell survival regulators, including glycogen synthase kinase‐3β (GSK‐3β) and calpain [36, 37]. Incorporation of Li^+^ into the EGCG nanocomplex likely potentiates these effects, thereby improving the resistance of ESC‐RGCs to oxidative stress‐induced apoptosis. Cell viability assays further validate these findings. Under oxidative conditions, both EGCG and Li‐EGCG NPs significantly improve ESC‐RGCs survival, with Li‐EGCG NPs demonstrating superior efficacy (Figure 4d), confirming the enhanced pro‐survival effect of Li‐EGCG NPs compared to EGCG alone.

To assess intracellular and mitochondrial oxidative stress, ROS levels were evaluated using CellROX (cytosolic ROS) and MitoSOX (mitochondrial superoxide) probes. Both EGCG and Li‐EGCG NPs treatments significantly reduce ROS accumulation (Figure 4e,n,o). However, Li‐EGCG NPs exhibit greater suppression of oxidative stress. As a potent polyphenolic antioxidant, EGCG has been widely reported to scavenge ROS and alleviate oxidative injury [27, 38, 39]. The enhanced efficacy of Li‐EGCG NPs likely results from a synergistic mechanism that combines the direct ROS‐scavenging capacity of EGCG with Li^+^ ability to modulate stress‐response signaling and suppress apoptosis.

To further investigate neuroprotection at the morphological level, we examined neurite integrity in ESC‐RGCs. After 5 days of culture to allow neural network formation, cells were subjected to H_2_O_2_ treatment and subsequently incubated with EGCG or Li‐EGCG NPs. The results reveal that Li‐EGCG NPs provide superior preservation of neurite morphology, as evidenced by larger soma area, increased neurite number, and thicker neurite diameter, compared to EGCG (Figure 4f,p,q; Figures S11 and S12). These findings are consistent with previous studies showing that EGCG can attenuate neurodegeneration by protecting against neurite loss and dendritic retraction [40]. Our data highlight that Li‐EGCG NPs further enhance this protective effect, offering superior maintenance of both neuronal structure and function under oxidative insult. Ultrastructural analysis using bio‐TEM further supported these observations (Figure 4g). ESC‐RGCs exposed to oxidative stress exhibit pronounced mitochondrial swelling and cristae disruption. In contrast, treatment with Li‐EGCG NPs confer marked protection, maintaining mitochondrial morphology and structural integrity, which shows a significant reduction in mitochondrial swelling and increased cristae density (Figure 4r,s).

In line with these structural improvements, a series of mitochondrial functional assays were performed to further assess mitochondrial integrity and bioenergetic status. Li‐EGCG NPs treatment significantly restored intracellular ATP production, indicating improved mitochondrial energy metabolism (Figure S13). In parallel, JC‐1 staining revealed a marked reduction of JC‐1 monomer formation in Li‐EGCG NPs treated group, suggesting preservation of mitochondrial membrane potential (Figure 4h,t). Moreover, MPTP assays showed that Li‐EGCG NPs effectively suppressed mitochondrial permeability transition pore opening, reflecting enhanced mitochondrial stability under oxidative stress (Figure 4i,u). Together, these results demonstrate that Li‐EGCG NPs preserve both the structural and functional integrity of mitochondria. These findings demonstrate that Li‐EGCG NPs confer robust neuroprotection in ESC‐RGCs by mitigating oxidative stress, inhibiting apoptosis, preserving mitochondrial structure and function, and maintaining neurite integrity. These results underscore the potential of Li‐EGCG NPs as a multifunctional therapeutic platform for retinal ganglion cell protection in glaucoma and as an adjunctive strategy to improve the survival and integration of transplanted ESC‐RGCs in cell replacement therapies.

### 2.5 Mechanistic Insights Into the Neuroprotective Effects of Li‐EGCG NPs

**FIGURE 5:**
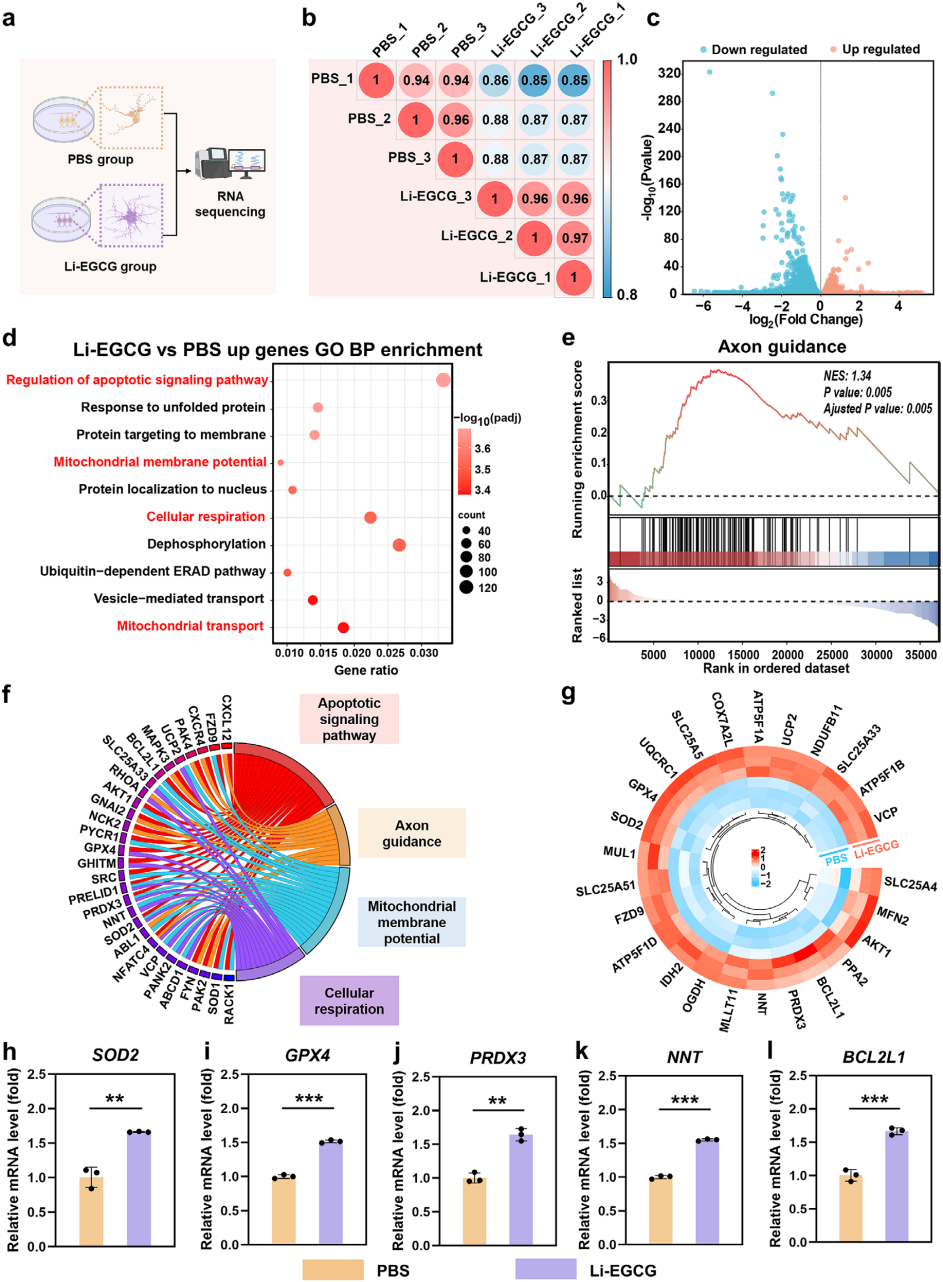
The molecular mechanisms underlying the neuroprotective effects of Li‐EGCG NPs. (a) Schematic overview of the transcriptomic analysis performed on ESC‐RGCs treated with PBS and Li‐EGCG NPs. (b) Correlation matrix plot demonstrating high intra‐group consistency and distinct inter‐group separation between the PBS and Li‐EGCG NPs‐treated groups. (c) Volcano plot illustrating DEGs. (d) GO enrichment analysis of upregulated genes in the Li‐EGCG NPs group. (e) GSEA highlighting activation of pathways involved in axon guidance. (f) Chord diagram summarizing key DEGs involved in neuroprotective pathways, including signaling pathway, axon guidance, mitochondrial membrane potential, and cellular respiration. (g) Heatmap showing upregulation of mitochondria‐related genes, including the antioxidant enzymes *SOD2*, *GPX4*, *PRDX3*, and *NNT*. (h–l) Quantitative analysis of the mRNA expression of *SOD2* (h), *GPX4* (i), *PRDX3* (j), *NNT* (k), and *BCL2L1* (l) in PBS and Li‐EGCG NPs‐treated groups (*n* = 3). Data are presented as mean ± SD; statistical analysis was performed using Student's *t*‐tests (^*^ *p* < 0.05, ^**^ *p* < 0.01 and ^***^ *p* < 0.001).

To elucidate the molecular mechanisms underlying the neuroprotective effects of Li‐EGCG NPs on ESC‐RGCs under oxidative stress, we performed transcriptomic profiling via RNA sequencing, followed by comprehensive bioinformatic analysis comparing Li‐EGCG NPs‐treated and PBS‐treated control groups (Figure 5a). Principal component analysis (PCA) demonstrates a clear segregation between the two groups, indicating that Li‐EGCG NPs induce substantial transcriptomic reprogramming in ESC‐RGCs (Figure S14). Consistent with this, the correlation matrix shows high intra‐group reproducibility, with a Pearson correlation coefficient of 1.0 within each group, confirming the robustness of the sequencing data (Figure 5b). Differential gene expression analysis identifies 4,411 significantly upregulated and 3,731 downregulated genes following Li‐EGCG NPs treatment (Figure 5c), indicating a broad and coordinated cellular response. Gene Ontology (GO) enrichment analysis of upregulated genes reveals significant enrichment in biological processes related to regulation of apoptotic signaling, maintenance of mitochondrial membrane potential, cellular respiration, and mitochondrial transport (Figure 5d). These processes are critical for neuronal survival and stress adaptation. Gene Set Enrichment Analysis (GSEA) further highlights positive enrichment of pathways associated with axon guidance and cellular metabolism (Figure 5e; Figure S15), suggesting a coordinated activation of neuroprotective and bioenergetic programs. To visualize functionally relevant gene expression changes, the chord diagram was constructed, summarizing key differentially expressed genes (DEGs) involved in neuroprotective pathways (Figure 5f). The heatmap shows robust upregulation of mitochondria‐associated antioxidant enzymes, including *SOD2*, *GPX4*, *PRDX3*, and *NNT*, which are well‐documented regulators of mitochondrial redox homeostasis and cell survival (Figure 5g) [41, 42]. The mRNA expression levels of cellular respiration (*SOD2*, *GPX4*, *PRDX3*, and *NNT*), apoptotic signaling pathway (*BCL2L1*, *CXCL12*, and *FZD9*), and axon guidance (*CXCR4*, *MAPK3*, and *PAK4*) related genes are also evaluated (Figure 5h–l; Figure S16). Notably, mitochondrial‐centered regulatory networks are highly relevant to glaucoma pathogenesis, as mitochondrial dysfunction is recognized as an upstream driver of retinal ganglion cell apoptosis and axonal degeneration, ultimately resulting in irreversible neuronal loss [29]. In this context, GPX4, a key regulator of mitochondrial lipid peroxidation and redox homeostasis, is significantly downregulated under glaucomatous conditions and is associated with pronounced mitochondrial ultrastructural abnormalities, including increased membrane density and disrupted cristae architecture. Restoration of GPX4 expression has been shown to suppress both apoptosis and ferroptosis while significantly promoting optic nerve regeneration, RGC survival, and visual function across multiple experimental models [43, 44, 45, 46]. Similarly, SOD2, a mitochondrial matrix‐localized superoxide dismutase, plays a pivotal role in detoxifying mitochondrial reactive oxygen species. Reduced SOD2 expression activates pro‐apoptotic signaling, characterized by elevated Bax levels and diminished Bcl‐xL expression, thereby increasing neuronal susceptibility to oxidative stress‐induced cell death [47]. The coordinated upregulation of mitochondrial antioxidant enzyme genes observed in the Li‐EGCG NPs group provides a mechanistic explanation for the suppression of apoptosis, preservation of mitochondrial function and maintenance of axonal integrity.

Kyoto Encyclopedia of Genes and Genomes (KEGG) pathway analysis further confirms the enrichment of signaling pathways such as axon guidance, neurotrophin signaling, and the cAMP signaling cascade, all known to support neuronal differentiation, neurite extension, and stress resistance (Figure S17). These results indicate that Li‐EGCG NPs exert their neuroprotective effects through modulated regulation of key cellular processes. Among these, the restoration and maintenance of mitochondrial homeostasis emerge as a central mechanism linking apoptosis inhibition with axonal preservation. This is consistent with a large body of literature identifying mitochondria as critical regulators of both neuronal survival and neurite development, especially under oxidative stress conditions [48, 49, 50]. Mitochondrial dysfunction, characterized by loss of membrane potential and cytochrome c release, is a hallmark of apoptosis initiation, while impaired ATP production and calcium buffering capacity directly compromise axonal maintenance and regenerative capacity [41, 51, 52].

In this study, Li‐EGCG NPs significantly preserved mitochondrial ultrastructure, upregulated antioxidant defense genes, and enhanced the expression of genes involved in mitochondrial energy metabolism and axonal signaling. These findings strongly support the notion that mitochondrial protection is a key driver of the dual anti‐apoptotic and neurite‐protective effects of Li‐EGCG NPs.

### 2.6 Restoration of Visual Function via Li‐EGCG@ESC‐RGCs Combination Therapy

Previous studies have consistently reported low survival rates of transplanted RGCs in vivo, with estimates often below 5% [12], largely due to hostile retinal microenvironments and limited neurite outgrowth from grafted cells. These challenges significantly restrict the efficacy of RGC‐based cell replacement therapies. To overcome these limitations, we developed a combinatorial strategy that integrates Li‐EGCG NPs with ESC‐RGCs. This Li‐EGCG@ESC‐RGCs formulation was designed to simultaneously promote the survival and functional integration of transplanted ESC‐RGCs and protect endogenous RGCs within an ischemia/reperfusion (I/R)‐induced acute pathological glaucomatous injury mouse model [30, 53]. To assess the systemic biosafety of Li‐EGCG@ESC‐RGCs therapy, we conducted the evaluation encompassing hematological analysis, serum biochemistry, and histopathological examination across four groups: I/R, ESC‐RGCs, EGCG@ESC‐RGCs and Li‐EGCG@ESC‐RGCs. Routine blood tests and biochemical parameters show no significant abnormalities (Figures S18 and S19), indicating no hematotoxicity or liver/kidney dysfunction. Moreover, histological examination of major organs, including heart, liver, spleen, lungs, and kidneys, reveals no signs of inflammation, necrosis or structural abnormalities in any group (Figure S20), demonstrating the favorable systemic biocompatibility of the Li‐EGCG@ESC‐RGCs therapeutic strategy.

**FIGURE 6:**
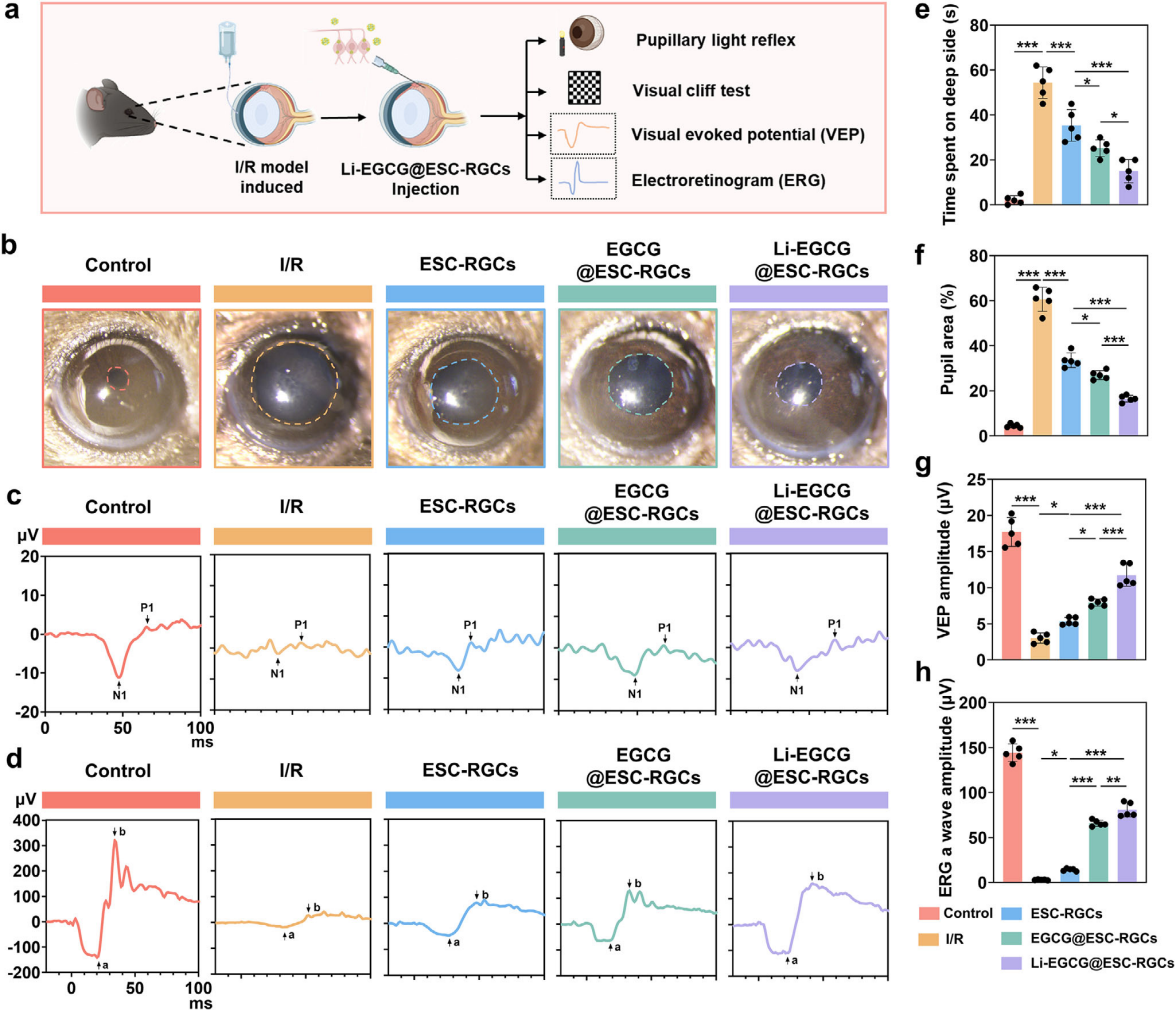
Restoration of visual function in I/R‐injured mice following treatment with Li‐EGCG@ESC‐RGCs. (a) Schematic illustration of the experimental design. (b) Representative images of PLR tests showing pupil constriction responses in each treatment group. (c) Representative traces of VEP waveforms recorded from the visual cortex. (d) Representative ERG waveforms demonstrating retinal response under low‐light conditions in each group. (e) Quantitative analysis of depth perception using the visual cliff test, as indicated by the percentage of time spent on the deep side (*n* = 5). (f) Quantification of pupil area measured during the PLR test (*n* = 5). (g) Quantification of VEP amplitudes across groups (*n* = 5). h) Quantitative analysis of ERG a‐wave amplitude (*n* = 5). Color coding: red (control), yellow (I/R), blue (ESC‐RGCs), green (EGCG@ESC‐RGCs), purple (Li–EGCG@ESC‐RGCs). Data are presented as mean ± SD; statistical analysis was performed using one‐way ANOVA followed by Tukey's post hoc tests (^*^ *p* < 0.05, ^**^ *p* < 0.01, ^***^ *p* < 0.001).

To evaluate visual function recovery, we performed a series of behavioral and electrophysiological tests (Figure 6a). Analysis of the pupillary light reflex reveals that I/R injury leads to evident pupillary dilation, indicative of impaired light‐responsive RGC activity (Figure 6b,f). Treatment with ESC‐RGCs or EGCG@ESC‐RGCs partially ameliorates this defect. Notably, Li‐EGCG@ESC‐RGCs‐treated mice exhibit significantly greater pupillary constriction, closely resembling the healthy control group, suggesting superior preservation or functional integration of RGCs. The visual cliff test, which assesses depth perception and visual cognition, further supports the efficacy of the combination therapy (Figure 6e; Figure S21). I/R mice exhibit marked deficits in depth perception, spending significantly more time on the safe platform. While both ESC‐RGCs and EGCG@ESC‐RGCs partially improve performance, the Li‐EGCG@ESC‐RGCs group demonstrates the most significant enhancement, indicating more robust recovery of visual signal processing and spatial discrimination. Electrophysiological recordings corroborate these behavioral findings. Visual evoked potentials (VEPs) show that I/R injury markedly reduced P1‐N1 amplitudes, reflecting attenuated cortical responses to visual stimuli (Figure 6c,g). Similarly, electroretinography (ERG) reveals substantial declines in a‐ and b‐wave amplitudes following I/R injury (Figure 6d,h; Figure S22), consistent with impaired retinal function. Treatment with ESC‐RGCs or EGCG@ESC‐RGCs partially restores these electrophysiological responses, while Li‐EGCG@ESC‐RGCs therapy leads to the most substantial recovery of both VEP and ERG signals. These results indicate that the combination of Li‐EGCG NPs with ESC‐RGCs confers enhanced neuroprotection and functional rescue of the visual pathway.

### 2.7 Retinal Morphological Preservation Mediated by Li‐EGCG@ESC‐RGCs Therapy

**FIGURE 7:**
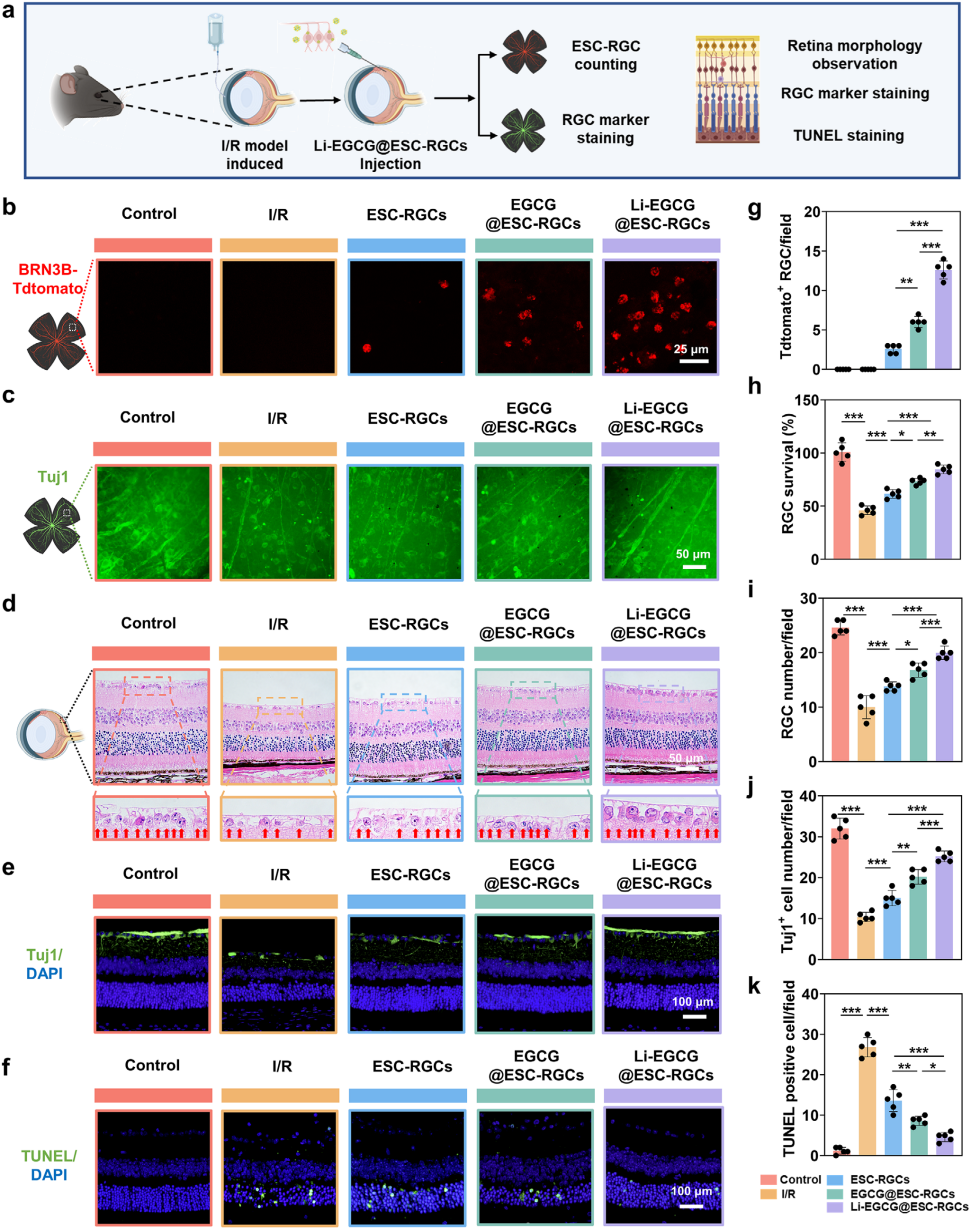
Recovery of retinal morphology in I/R mice following Li‐EGCG@ESC‐RGCs treatment. (a) Schematic overview of the experimental procedure. (b) Retinal flat‐mounts showing red fluorescence‐labeled ESC‐RGCs imaged by confocal microscopy. (c) Retinal flat‐mounts stained for Tuj1^+^ cells (green fluorescence). (d) H&E staining of retinal cross‐sections from each group. Enlarged views of the ganglion cell layer (GCL) highlight RGC nuclei, marked by red arrows. (e) Immunofluorescence staining of retinal sections showing the expression of Tuj1 (green fluorescence) in different treatment groups. (f) TUNEL staining of retinal sections displaying apoptotic cells (green fluorescence) across different treatment groups. (g) Quantification of Tdtomato^+^ RGCs in different treatment groups (*n* = 5). (h) Quantification of Tuj1^+^ cells in different treatment groups (*n* = 5). (i) Quantification of RGC numbers based on H&E‐stained sections (*n* = 5). (j) Quantification of Tuj1^+^ cell numbers based on retinal sections in different treatment groups (*n* = 5). (k) Quantification of TUNEL‐positive cells in different treatment groups (*n* = 5). Red (control), yellow (I/R), blue (ESC‐RGCs), green (EGCG@ESC‐RGCs), purple (Li‐EGCG@ESC‐RGCs). Data are presented as mean ± SD; statistical analysis was performed using one‐way ANOVA followed by Tukey's post hoc tests (^*^ *p* < 0.05, ^**^ *p* < 0.01 and ^***^ *p* < 0.001).

To further investigate the in vivo neuroprotective effects of Li‐EGCG NPs on RGCs, we assessed transplanted cell survival, RGC‐specific marker expression, retinal structural integrity, and apoptosis in a murine model of I/R‐induced retinal injury (Figure 7a). The results reveal a significantly higher number of TdTomato‐positive cells in retinas treated with Li‐EGCG@ESC‐RGCs compared to the ESC‐RGCs and EGCG@ESC‐RGCs groups (Figure 7b,g), indicating enhanced survival and retention of grafted cells facilitated by Li‐EGCG NPs. Furthermore, future strategies such as internal limiting membrane digestion may further improve the survival rate of transplanted cells. Moreover, immunofluorescence staining for Tuj1 a pan‐neuronal marker, demonstrates more robust neurite‐like projections and increased cell counts in the Li‐EGCG@ESC‐RGCs group (Figure 7c,h), suggesting improved viability and neurite protection of RGCs. Hematoxylin and eosin (H&E) staining further confirms superior preservation of retinal architecture, with a higher density of ganglion cell layer neurons in the Li‐EGCG@ESC‐RGCs group compared to other treatments (Figure 7d,i). Consistent with this finding, cross‐sectional immunostaining of retinal layers demonstrates a significantly higher number of Tuj1‐positive cells in the Li‐EGCG@ESC‐RGCs group, supporting the conclusion that this combinatorial therapy enhances RGC survival at the structural level (Figure 7e,j). Apoptotic cell death, as assessed by TUNEL staining, is markedly attenuated following Li‐EGCG@ESC‐RGCs treatment (Figure 7f), with quantification revealing a significant reduction in TUNEL‐positive cells compared to the ESC‐RGCs and EGCG@ESC‐RGCs groups (Figure 7k). These results suggest that Li‐EGCG NPs confer robust anti‐apoptotic effects, likely mediated by their antioxidative capacity and mitochondrial‐protective mechanisms.

Li‐EGCG NPs not only improve the survival and structural integration of transplanted ESC‐RGCs but also mitigate I/R‐induced apoptosis and degeneration of host RGCs, thus providing dual protection within the damaged retina. Previous studies have explored nanotechnology‐based approaches to preserve endogenous RGCs in glaucoma models. For instance, HOLN‐NPs, a dual‐responsive nanoplatform co‐delivering nicotinamide and oleic acid, was shown to reduce oxidative stress and activate the CaMKII/CREB axis, thereby maintaining mitochondrial integrity in RGCs [22]. Similarly, a ROS‐responsive nanoparticle loaded with necrostatin‐1, effectively inhibited necroptosis and limited RGC loss under glaucomatous stress [53]. However, these strategies primarily focused on preserving endogenous RGCs and did not address the persistent challenges associated with cell replacement therapies, including poor graft survival and limited integration. In contrast, our Li‐EGCG NPs based approach offers a dual‐action solution by simultaneously protecting host neurons and enhancing the survival and functionality of transplanted ESC‐RGCs. This synergistic effect, combining cytoprotection, anti‐apoptotic modulation, and neurite preservation, positions Li‐EGCG NPs as a promising nanotherapeutic platform for advancing cell‐based interventions in glaucomatous optic neuropathies. By bridging regenerative cell replacement with neuroprotective modulation, our work provides a conceptual and experimental foundation for combinatorial therapeutic strategies targeting neuroretinal degenerative diseases.

Nevertheless, several challenges remain for the clinical translation of ESC‐RGC‐based therapies. These include ethical considerations associated with embryonic stem cell sources and the high cost of producing clinical‐grade ESCs or ESC‐derived cells suitable for human transplantation [54, 55]. In addition, the present study was conducted in a mouse model using intravitreal administration, and further investigations in non‐human primate models are required to optimize dosing, delivery routes and therapeutic windows prior to clinical translation [56, 57]. Long‐term follow‐up studies will also be essential to rigorously evaluate the safety, durability, and sustained therapeutic efficacy of this strategy [58]. Despite these limitations, our findings highlight the significant translational potential of Li‐EGCG NPs as a multifunctional adjunct to RGC replacement therapies for optic nerve and retinal neurodegenerative disorders.

## 3 Conclusion

In summary, we have engineered a distinct Li‐EGCG NPs system, integrating lithium ions with EGCG into a stable nanocomplex that demonstrates potent broad‐spectrum antioxidant activity and enhanced neuroprotective efficacy. Compared to free EGCG, Li‐EGCG NPs feature superior protection against oxidative stress‐induced apoptosis and neurite degeneration in ESC‐RGCs, while promoting mitochondrial integrity and functional recovery in vivo. In a retinal ischemia/reperfusion injury acute pathological glaucomatous injury model, the combinatorial therapy of Li‐EGCG NPs and ESC‐RGCs transplantation significantly improves cell survival, neurite preservation, and visual function restoration. These findings underscore the robust synergistic benefits of Li‐EGCG NPs in supporting both endogenous and transplanted retinal ganglion cells, presenting a promising nanomedicine strategy to advance cell‐based therapeutic approaches for retinal neurodegenerative disorders.

## 4 Experimental Section

### 4.1 Preparation of the Li‐EGCG NPs

The LiCl·H_2_O and EGCG were used in this study to prepare the Li‐EGCG NPs. LiCl·H_2_O and EGCG in a molar proportion of 4:1 were dissolved in ethanol to obtain the final concentrations of 4 and 1 mmol/L. After complete dissolution, the pH of the solution was carefully adjusted to 7.0 using sodium carbonate. The resulting mixture was maintained at 37°C under constant stirring for 24 h to promote the formation of the Li‐EGCG NPs. Following the reaction, the suspension was subjected to centrifugation to collect the product, which was subsequently washed multiple times with ethanol. The purified Li‐EGCG NPs appeared as a light brown powder after vacuum drying.

### 4.2 Characterization of the Li‐EGCG NPs

Transmission electron microscopy (TEM) was carried out using a JEOL JEM‐F200 transmission electron microscope (Japan) to observe the morphology and perform element mapping of the Li‐EGCG NPs. Scanning electron microscopy (SEM) images were captured by a JEOL JSM‐6700F scanning electron microscope (Japan). Dynamic light scattering (DLS) analysis was performed using a Zetasizer Nano ZSE instrument (Malvern Instruments, UK) to determine the hydrodynamic diameter of the Li‐EGCG NPs. The morphology of Li‐EGCG NPs was examined using an atomic force microscope (AFM, Bruker Dimension Icon, Germany). Fourier transform infrared (FTIR) spectroscopy was performed using a SHIMADZU IRTracer‐100 spectrometer (Japan). Data were collected over the range of 4000–400 cm^−^^1^ with a resolution of 4 cm^−^^1^ and 32 scans. Chemical composition analysis was conducted using X‐ray photoelectron spectroscopy (XPS, ESCALAB 250Xi, Thermo Fisher Scientific, USA). UV–vis absorption spectra were obtained by using a UV‐1800 spectrophotometer (MAPADA, China). Electron spin resonance (ESR) spectra were acquired on an EMXplus spectrometer (Bruker, Germany).

### 4.3 Evaluation of ABTS·+ Scavenging Activity

To perform the assay, 0.2 mL of 2,2’‐azino‐bis (3‐ethylbenzothiazoline‐6‐sulfonic acid) (ABTS) solution (7.4 mmol/L) was mixed with 0.2 mL of potassium persulfate (K_2_S_2_O_8_) solution (2.6 mmol/L) and incubated in the dark overnight to form ABTS·+ radicals. The resulting solution was then diluted with PBS. Subsequently, 1.8 mL of Li‐EGCG NPs solutions at different concentrations (0, 25, 50, 75, and 100 µg/mL) were mixed with 0.20 mL of ABTS·+ solution. After incubation, the absorbance at 734 nm was measured using a UV–vis spectrophotometer. As shown in Figure 2b,c, the absorbance decreased with increasing concentrations of Li‐EGCG NPs, indicating enhanced ABTS·+ scavenging capacity and improved antioxidant activity. The detailed evaluation methods for DPPH·,·OH,·O_2_^−^, and H_2_O_2_ scavenging activity are provided in the Supporting Information.

### 4.4 ESC‐RGCs Differentiation

The ESC‐RGCs differentiation was induced by adding different sets of small molecules into the media at specific stages (Figure 3a), according to the previously published protocols [14]. Briefly, at DAY ‐1, ESCs were passaged by TrypLE, and the suspension of 50.5 × 10^4^ single cells was seeded to a 6‐well plate. At DAY 0, the media was changed to N2B27 media, which contains 1:1 mix of DMEM/F12 and Neurobasal with 1× GlutaMAX Supplement, 1% penicillin‐streptomycin, 1% N‐2 Supplement, and 2% B‐27 Supplement. At DAY 1–6, 25 µm Forskolin, 2.5 µm IDE2, 10 mm Nicotinamide, and 1 µm Dorsomorphin were added to the N2B27 media. At DAY 7–10, 25 µm Forskolin and 10 mm Nicotinamide were added to the N2B27 media. At DAY 11–18, 25 µm Forskolin was added to the N2B27 media. After DAY 19, 25 µm Forskolin and 10 µm DAPT were added to the N2B27 media. The culture medium was replaced daily. Red fluorescence was observed in ESC‐derived RGCs by DAY 17, with cells reaching maturation by DAY 28. Cell images were captured using a fluorescence microscope (Nikon, Japan) at an emission wavelength of 594 nm for red fluorescence, and bright‐field images were acquired using the same microscope.

### 4.5 Immunofluorescence Staining

The ESC‐RGCs were seeded onto 24‐well plates and untreated for Tuj1, Islet1 and RBPMS staining. Then cells were fixed with 4% paraformaldehyde (PFA) for 10 min at room temperature. After washing with PBS, the cells were blocked with immunol staining blocking buffer for 1 h. Then the cells were incubated overnight at 4°C with the following primary antibodies: anti‐mouse Tuj1 antibody (1: 200), anti‐mouse Islet1 antibody (1:200), and anti‐rabbit RBPMS antibody (1: 200). After washing with PBS, the cells were incubated for 1 h at room temperature with appropriate secondary antibodies: anti‐mouse Alexa Fluor 488 IgG (1:600) or anti‐rabbit Alexa Fluor 488 IgG (1:600). Nuclei were counterstained with DAPI before imaging. Images were acquired using a fluorescence microscope (Nikon, Japan).

For retinal whole‐mount preparation, the eyeballs of mice in different groups were fixed in 4% PFA at 4°C overnight. Then the retina was carefully isolated, flattened, and mounted onto glass slides with the ganglion cell layer facing upward. For TdTomato‐BRN3B^+^ ESC‐RGCs, observing samples were mounted in antifade medium and imaged using a confocal laser scanning microscope (ZEISS LSM 880, Germany). Blocking was performed with an immunol staining blocking buffer for 1 h. Retina were incubated overnight at 4 °C with anti‐mouse Tuj1 antibody (1:200). After washing with PBS, tissues were incubated with anti‐mouse Alexa Fluor 488 IgG (1:600) and counterstained with DAPI. Additionally, for paraffin‐embedded retinal sections, after dewaxing and rehydration, tissue sections were blocked with the same blocking buffer and stained with anti‐mouse Tuj1 antibody using the same immunostaining protocol as above.

### 4.6 TUNEL Assay

First, different treatment (EGCG, Li‐EGCG NPs) at 25 µg/mL was given to the H_2_O_2_‐treated ESC‐RGCs. Then, we used the terminal deoxynucleotidyl transferase dUTP nick end labeling (TUNEL) staining by a one‐step TUNEL Apoptosis Assay Kit (Beyotime, China) to determine cell apoptosis, investigating the protective effects of EGCG and Li‐EGCG NPs. After washing with PBS three times, a mixture of TUNEL detection solution (TdT enzyme and fluorescent labeling solution in a 1:9 ratio) was added to each sample and incubated for 1 h in the dark. Following another three PBS washes, nuclei were counterstained with DAPI. Images were then captured using a fluorescence microscope (Nikon, Japan) at emission wavelengths of 405 and 488 nm.

To evaluate apoptosis levels in the retina across different groups, a TUNEL assay was also performed on retinal sections. After dewaxing and rehydration, proteinase K (20 µg/mL) was applied to the sections and incubated at 37°C for 15 min. The subsequent procedures were carried out following the same protocol as described above.

### 4.7 Animals

All animal procedures in this study were conducted in compliance with the Association for Research in Vision and Ophthalmology (ARVO) Statement for the use of animals in ophthalmic and vision research, and received approval from the Institutional Animal Care Committee of Ninth People's Hospital, School of Medicine, Shanghai Jiao Tong University (Approval No. SH9H‐2023‐A93‐1). Male C57BL/6J mice (6–8 weeks old, 20–25 g) were obtained from JieSiJie Laboratory Animal Co., Ltd. (Shanghai, China) and maintained under specific pathogen‐free (SPF) conditions.

For visual evoked potential (VEP) and electroretinography (ERG), mice were anesthetized using isoflurane (RWD Life Science, China) delivered via a small animal anesthesia machine (Shanghai YuYan Instruments, China). Anesthesia induction was performed for 3 min per mouse with 3.0% isoflurane in 1.0 L/min oxygen, followed by maintenance for 25 min at 1.5% isoflurane with a 0.6 L/min oxygen flow. For all other experiments in this study, anesthesia was achieved through intraperitoneal injection of tribromoethanol (0.2 mL/10 g; Nanjing Aibei Biotechnology Co., Ltd., China).

### 4.8 Retinal Ischemia/Reperfusion (I/R) Injury

The induction of retinal ischemia‐reperfusion (I/R) acute pathological glaucomatous injury model was according to the previous reported literature [59]. Briefly, mice were first anesthetized using tribromoethanol. Then, a 30‐gauge needle connected to a 120 cm column of sterile isotonic saline was inserted into the anterior chamber of the right eye for 60 min, as illustrated in Figures 6a, 7a. The intraocular pressure (IOP) was monitored during the procedure using the Icare TonoLab tonometer (Icare Finland Oy, Finland), to assure the IOP of the operated eye was elevated from approximately 10 to 90 mmHg. Mice that did not undergo the I/R procedure served as the control group. In the PBS, ESC‐RGCs, EGCG@ESC‐RGCs, Li‐EGCG@ESC‐RGCs group, 2 µL of PBS, ESC‐RGCs (2 × 10^4^), EGCG@ESC‐RGCs (2×10^4^ ESC‐RGCs with EGCG) and Li‐EGCG@ESC‐RGCs (2×10^4^ ESC‐RGCs with Li‐EGCG NPs) were injected through a 5 µL microliter syringe with 33G needle (Hamilton, Germany) into the right eye of each mouse through intravitreal injection, after the day of I/R model induction. Animals were euthanized at 7 days post‐injury for subsequent analyses.

### 4.9 Visual Function Assessment in Mice

To comprehensively evaluate visual function in mice, a series of behavioral and electrophysiological tests were conducted, including pupillary light reflex, visual cliff test, visual evoked potential (VEP), and electroretinography (ERG).

The pupillary light reflex was assessed by exposing dark‐adapted mice of different groups to a bright light stimulus and recording pupil constriction using an infrared camera. In addition, visual cliff tests were conducted to evaluate depth perception, in which each mouse was placed on a platform with an apparent drop‐off, the non‐model eye was dilated using mydriatic eye drops, and their movement preference toward the shallow or deep side was recorded.

For electrophysiological tests, after 12 h of dark adaptation, mice were anesthetized with isoflurane using a small animal anesthesia system, as described above. Pupils were dilated using topical mydriatic agents. The electrodes were placed on the corneas, the subcutaneous tissue of the head, and the back for ERG, and subcutaneously over the visual cortex, with reference positioned anteriorly and ground electrodes on the back for VEP. The scotopic ERG responses (10.0 photons/µm^2^) and VEP signals were recorded using the Espion E3 system (Diagnosys LLC., Germany) to evaluate retinal and cortical visual function, following previously published protocols [60].

### 4.10 H&E Staining

At 7 days after injury, mice were euthanized, and the eyeballs, along with major organs including the heart, liver, spleen, lung, and kidney, were collected for histological analysis. Eyeballs were fixed in FAS fixative solution (Servicebio, China), while the remaining tissues were fixed in 4% paraformaldehyde. Following fixation, tissues underwent graded alcohol dehydration, paraffin embedding, and were sectioned into 5 µm slices. For hematoxylin and eosin (H&E) staining, sections were dewaxed and rehydrated, then stained with hematoxylin for 8 min. After rinsing in running water, sections were differentiated in 0.7% hydrochloric acid ethanol for 2 s, followed by bluing under running water for 20 min. Slides were then immersed in 95% ethanol for 1 min, stained with eosin for 3 min, passed again through 95% ethanol (1 min, twice), 100% ethanol (1 min, twice) and cleared with xylene I and II (3 min each). Images were acquired using a Nikon microscope (Japan), and RGC numbers were quantified at a standardized distance of 400 µm from the optic nerve head.

### 4.11 Complete Blood Count and Serum Biochemistry

Blood samples were collected from mice via retro‐orbital puncture under isoflurane anesthesia. Whole blood was collected in EDTA‐coated tubes for complete blood count, and in anticoagulant‐free tubes for serum biochemistry. After clotting at room temperature and centrifugation at 3000 rpm for 10 min, serum was separated. All samples were submitted to Servicebio (Wuhan, China) for analysis. The complete blood count was performed on whole blood, while serum was used for biochemical tests including ALT, AST, ALB, ALP, TP, BUN, TG and LDH.

### 4.12 Statistical Analysis

In this study, all quantitative data obtained from independent in vitro and in vivo experiments are presented as means ± standard deviations (SDs), with statistical analyses performed using SPSS Statistics 27 (USA). Comparisons between two groups were performed using unpaired two‐tailed Student's *t*‐tests when data were normally distributed. One‐way analysis of variance (ANOVA) was used to assess differences among groups, followed by Tukey's multiple comparisons test as a post hoc analysis to identify significant pairwise differences in three or more groups. Statistical graphs were generated using GraphPad Prism version 10.0 (USA) to visually present the results. A *p*‐value of ^*^ *p* < 0.05, ^**^ *p* < 0.01, and ^***^ *p* < 0.001 was considered indicative of statistical significance.

## Author Contributions

P.G., Y.C., and W.F. conceived the study. P.G., Y.C., W.F., N.N., M.C., X.J. and Y.W. designed the experiments. M.C., X.J., Y.W., Z.L., Y.Z., S.L., R.H., N.N., D.Z., Z.T., Y.J. and S.P. performed experiments and analyzed data. P.G., Y.C., W.F. and N.N. supervised experiments and data analysis. P.G., Y.C., W.F., M.C., X.J. and Y.W. wrote the manuscript.

## Conflicts of Interest

The authors declare no conflicts of interest.

## Supporting information




**Supporting File**: advs74509‐sup‐0001‐SuppMat.docx

## Data Availability

The data that support the findings of this study are available from the corresponding author upon reasonable request.
