# Supplementary material for: Anti‐Apoptotic and Neurite‐Protective Nanomedicine Augments Embryonic Stem Cells‐Derived Retinal Ganglion Cell Transplantation in Glaucoma Recovery
Source: Adv Sci (Weinh). 2026 Feb 21;13(25):e13499. doi: 10.1002/advs.202513499 (PMC13137833; doi:10.1002/advs.202513499)
Supplement: Supplementary file 1 — Supporting File: advs74509‐sup‐0001‐SuppMat.docx [file ADVS-13-e13499-s001.docx]

Supporting Information

**Anti-apoptotic and Neurite-protective Nanomedicine Augments Embryonic Stem Cells-Derived Retinal Ganglion Cell Transplantation in Glaucoma Recovery**

*Moxin Chen^#^, Xiaoyan Jiang^#^, Yuanhui Wang^#^, Zhaobin Luo, Yu Zhang, Siwei Liu, Rui Huang, Dandan Zhang, Zhimin Tang, Yahan Ju, Shaohui Pan, Ni Ni^*^, Wei Feng^*^, Yu Chen^*^, Ping Gu^*^*

M. Chen, Y. Wang, Z. Luo, Y. Zhang, S. Liu, R. Huang, D. Zhang, Z. Tang, Y. Ju, N. Ni, Prof. P. Gu

Department of Ophthalmology, Ninth People’s Hospital, Shanghai Jiao Tong University School of Medicine, Shanghai 200011, P. R. China.

Shanghai Key Laboratory of Orbital Diseases and Ocular Oncology, Shanghai 200011, P. R. China.

Email: 1127239413@sjtu.edu.cn (N. Ni); guping2009@sjtu.edu.cn (Prof. P. Gu)

Prof. W. Feng, Prof. Y. Chen

Materdicine Lab, School of Life Sciences, Shanghai University, Shanghai 200444, P. R. China

Email: fengw@shu.edu.cn (Prof. W. Feng); chenyuedu@shu.edu.cn (Prof. Y. Chen)

X. Jiang

Materdicine Lab, School of Life Sciences, Shanghai University, Shanghai 200444, P. R. China

School of Medicine, Shanghai University, Shanghai, P. R. China

Prof. S. Pan

State Key Laboratory of Eye Health, Eye Hospital, Wenzhou Medical University, Wenzhou, 325027, P. R. China.

^*^Corresponding authors.

^#^These authors contributed equally to this work.

**1. Supplementary Experimental Section**

***Materials*:** LiCl·H_2_O (MW60.41 g/mol), epigallocatechin gallate (EGCG, MW458.37 g/mol), titanium sulfate [Ti(SO_4_)_2_], 2,2’-azino-bis (3-ethylbenzothiazoline-6-sulfonic acid) (ABTS), sodium salicylate (NaSal), ferrous sulfate (FeSO_4_), and DPPH were purchased from Macklin lnc., China. Hydrochloric acid (HCl), hydrogen peroxide (H_2_O_2_), sulfuric acid (H_2_SO_4_), and ethanol were purchased from Sinopharm Chemical Reagent Co., China. The ·O_2_^-^ scavenging reagent, immunol staining blocking buffer, DAPI, and one Step TUNEL Apoptosis Assay Kit were purchased from beyotime Biotech lnc, China. TeSR-E8 Basal Medium, ReLeSR, and IDE2 were purchased from STEM CELL, Canada. Matrigel was purchased from Corning, USA. Y-27632 2HCl was purchased from Selleck chemicals, UK. High-glucose and low-glucose Dulbecco’s Modified Eagle Medium (DMEM), fetal bovine serum (FBS) for ARPE-19 and rRMC cells, penicillin-streptomycin, TrypLE, DMEM/F12, Neurobasal with 1X GlutaMAX Supplement, N-2 Supplement, B-27 Supplement, and 0.25% trypsin-EDTA were purchased from Gibco, USA. Fetal bovine serum (FBS) for 661W cells, Forskolin, Nicotinamide, Dorsomorphin, and DAPT were purchased from Sigma, USA. Type II Collagenase was purchased from Worthington Biochemical Corp., USA. Cell Counting Kit-8 (CCK8) was purchased from Yeasen Biotechnology, China. Live/dead staining kit, CellRox kit, and MitoSox kit was purchased from Invitrogen, USA. The 4% paraformaldehyde (PFA) was purchased from Biosharp, China. The primary antibodies for immunofluorescence (IF) staining included the anti-mouse Tuj1 antibody (Cat#801202, Biolegend, RRID: AB_2313773), anti-mouse Islet 1 antibody (Cat#Ab86501, Abcam, RRID: AB_1951289), and anti-rabbit RBPMS antibody (Cat#1830-RBPMS, Phosphosolutions, RRID: AB_2492225). The second antibodies for IF staining included anti-mouse Alexa Fluor 488 IgG (Cat#A-11001, Invitrogen, RRID: AB_2534069) or anti-rabbit Alexa Fluor 488 IgG (Cat#A-11008, Invitrogen, RRID: AB_143165).

***Evaluation of*** ***DPPH***· ***scavenging activity*:** For the DPPH· assay, a 0.04 mg/mL DPPH solution in ethanol was freshly prepared. Subsequently, 2 mL of this solution was mixed with 2 mL of Li-EGCG NPs solutions at different concentrations (0, 25, 50, 100, 200, and 400 μg/mL) and incubated for 30 minutes. The absorbance at 519 nm was then measured using a UV-vis spectrophotometer. As the concentration of Li-EGCG NPs increased, a gradual decrease in absorbance was observed (**Figure 2e,f**), suggesting an enhanced ability to scavenge DPPH· radicals and improved antioxidant activity.

***Evaluation of*** ·***OH radical scavenging activity*:** To evaluate the hydroxyl radical (·OH) scavenging activity of Li-EGCG, the salicylic acid (SA) method was employed. Hydroxyl radicals (·OH) are highly reactive and can specifically react with salicylic acid to produce 2,3-dihydroxybenzoic acid (2,3-DHBA) and 2,5-dihydroxybenzoic acid (2,5-DHBA). These products exhibit characteristic absorption peaks at specific wavelengths (typically around 510 nm), and by measuring their absorbance, the amount of ·OH generated in the system can be indirectly determined. Firstly, mixing 0.1 mL FeSO_4_ (2 mM) and 0.1 mL H_2_O_2_ (5 mM) and incubating for 5 minutes. Subsequently, 1.7 mL of Li-EGCG NPs solutions at different concentrations (0, 25, 50, 100, 200, and 400 μg/mL) were introduced into the reaction system, then 0.1 mL of salicylic acid ethanol solution (10 mM) was added, and the entire solution was thoroughly mixed and allowed to react for an additional 15 minutes. The remaining ·OH was quantified by monitoring the absorbance at 510 nm, corresponding to 2,3-dihydroxybenzoic acid, the oxidation product of SA by ·OH, using UV-vis spectroscopy. A concentration-dependent decrease in absorbance was observed with increasing Li-EGCG NPs levels (**Figure 2h,i**), suggesting enhanced scavenging of ·OH and improved overall antioxidant performance.

***Evaluation of*** ·***O_2_^-^ radical scavenging activity*:** The ability of Li-EGCG NPs to scavenge superoxide anions (·O_2_^-^) was assessed using a total antioxidant capacity assay kit based on the nitroblue tetrazolium (NBT) method (Beyotime, China), following the manufacturer’s protocol. The absorbance was recorded at 560 nm using a microplate reader. A concentration-dependent enhancement at different concentrations of 12.5, 25, 50, 100, and 200 μg/mL in superoxide anion scavenging efficiency was observed in **Figure 2l**, indicating improved antioxidant performance with increasing concentrations of Li-EGCG NPs.

***Evaluation of*** ***H_2_O_2_*** ***scavenging activity*:** Titanium sulfate spectrophotometric method (TSS) is a classical colorimetric method for the detection of hydrogen peroxide (H_2_O_2_). In this assay, titanium sulfate reacts with H_2_O_2_ to form a yellow peroxytitanium complex, which is soluble in strong acidic solution and exhibits a characteristic absorbance peak at 410 nm. The intensity of absorbance, which can be measured by UV-vis spectrophotometry, is linearly correlated with the concentration of H_2_O_2_ within a certain range. To prepare the H_2_O_2_ detection solution, 1.25 g of Ti(SO_4_)_2_ was dissolved in 8 mL of H_2_SO_4_ and diluted with deionized water to a final volume of 100 mL. Then, 200 μL of H_2_O_2_ (10 mM) was mixed with 1.2 mL of the detection solution, followed by the addition of 2 mL of Li-EGCG solution at various concentrations (0, 25, 50, 100, and 200 μg/mL). After incubation for 1 hour, the absorbance at 410 nm was recorded. As shown in **Figure 2n,o**, the absorbance decreased with increasing concentrations of Li-EGCG, indicating enhanced total antioxidant capacity and effective scavenging of H_2_O_2_.

***Cell culture*:** The human embryonic stem cells (ESCs) (RRID: CVCL_9773) were a kind gift from Eye Hospital, Wenzhou Medical University, and were obtained in November 2023.^[1]^ The human retinal pigment epithelial cell line ARPE-19 (RRID: CVCL_0145) was obtained from the Cell Bank, Chinese Academy of Sciences in June 2021. The 661W cell line (RRID: CVCL_6240), which originates from mouse photoreceptors, was generously provided by Dr. Al-Ubaidi from the University of Oklahoma in September 2021. Additionally, the rat Müller glial cell line rRMC (RRID: CVCL_8140) was kindly supplied by the Department of Ophthalmology at Shanghai General Hospital, affiliated with Shanghai Jiao Tong University in September 2023. All cell lines used in this study were regularly tested and confirmed to be free of mycoplasma contamination and other potential contaminants.

For cell culture, ESCs were maintained in TeSR-E8 Basal Medium with Matrigel plated, and 10 mM ROCK inhibitor Y-27632 2HCl was added after passaging by ReLeSR. A tdTomato reporter was inserted under the control of the *BRN3B* promoter, enabling the ESCs to express red fluorescence upon differentiation into retinal ganglion cells (RGCs) and activation of BRN3B expression. ARPE-19 cells were maintained in high-glucose Dulbecco’s Modified Eagle Medium (DMEM) supplemented with 10% fetal bovine serum (FBS) and 1% penicillin-streptomycin. The 661W cells were cultured in a similar high-glucose DMEM base, but with 10% Australian FBS and 1% penicillin-streptomycin. In contrast, rRMC cells were grown in low-glucose DMEM with the addition of 10% FBS and 1% penicillin-streptomycin.

All cell cultures were incubated under standard conditions of 37 °C with a humidified atmosphere containing 5% CO_2_.

***Cell viability detection*:** The cell viability was detected with Cell Counting Kit-8 (CCK-8) and live/dead staining kit. For CCK-8 test, the ESC-RGCs were digested with Type II Collagenase and suspended equally into 96-well plates. In **Figure 3d,e**, after 24- and 48-hours treatment with different concentrations (0, 5, 10, 25, 50, 100, 200, and 400 μg/mL) of EGCG and Li-EGCG NPs. In **Figure 4h**, cells were treated with 100 μM H_2_O_2_, then EGCG and Li-EGCG NPs at 25 μg/mL. Subsequently, the cells were incubated with CCK-8 for 4 hours, and tested for the optical density (O.D.) value at a wavelength of 450 nm by a microplate reader (Agilent, BioTek Epoch 2, USA). The cell viability value in CCK-8 assay was defined as the optical density of the treated group compared with the control group.

For live/dead staining, the ARPE-19, 661W, and rRMC cells were digested with 0.25% trypsin-EDTA and suspended equally into 24 wells. After 48 hours treatment with 25 μg/mL of EGCG and Li-EGCG NPs, the cells were stained 10 minutes with Calcein-AM (1:1000) for labeling live cells, and PI (1:1000) for labeling dead cells. After washing with PBS for 3 times, the cells images were taken under a fluorescence microscope (Nikon, Japan) at the emission wavelength of 488 nm and 594 nm.

For direct observation of cell morphology and red fluorescence in **Figure 3f,g**, ESC-RGCs were treated with EGCG and Li-EGCG NPs at a concentration of 25 μg/mL for 24 and 48 hours. Subsequently, fluorescence and bright-field images were acquired using a Nikon fluorescence microscope (Japan), with red fluorescence detected at an emission wavelength of 594 nm.

***Cellular and mitochondrial reactive oxygen species (ROS) detection*:** The cellular and mitochondrial ROS detection were using the CellRox kit and MitoSox kit from Invitrogen, USA, respectively. ESC-RGCs were exposed to H_2_O_2_ to induce oxidative stress, then treated with 25 μg/mL of EGCG, and different concentrations of Li-EGCG NPs (5, 10, 25, 50, 100, 200, and 400 μg/mL) for 24 hours. According to the manufacturer’s protocol, ESC-RGCs with different treatment were incubated with 5 μM CellRox or 1 μM MitoSox reagent for 30 minutes at 37 °C, then the cells were washed gently with washing buffer. Images were then captured using a fluorescence microscope (Nikon, Japan) at emission wavelengths of 488 nm.

***Neurite morphology analysis*:** To facilitate the observation of neurites, ESC-RGCs were digested with Type II collagenase and evenly seeded into 24-well plates. Cells were cultured for 5 days to allow the formation of neural networks. Subsequently, H_2_O_2_ treated cells were applied with 25 μg/mL of either EGCG or Li-EGCG NPs. The soma area, neurite number, and neurite diameter were quantified using ImageJ software (version 1.54, National Institutes of Health, USA).

***Mitochondrial Ultrastructure Analysis*:** The ultrastructural features (particularly mitochondrial morphology and integrity) of ESC-RGCs with different treatments were examined using TEM. After collection, the cells were fixed in 2.5% glutaraldehyde at 4°C, then rinsed with PBS. A graded ethanol series was used for dehydration, followed by infiltration with propylene oxide and embedding in Epon 812 resin. Ultrathin sections were cut using a LEICA EM UC7 ultramicrotome (Germany), stained with uranyl acetate and lead citrate, and subsequently observed under a Thermo Fisher Scientific Inc. FEI Talos L120C transmission electron microscope (USA). The relative mitochondrial size and cristae number/mito area were quantified using ImageJ software (version 1.54, National Institutes of Health, USA).

***Western Blot*:** A total of 100 μL of RIPA buffer mixed with 1 μL of protease and phosphatase inhibitor was added to cells with different treatments, incubated on ice for 10 minutes, and then centrifuged at 12000 rpm for 15 minutes at 4 °C. After ultrasonication to break up the pellet, the sample was added with 20 μL of 5× reducing sample buffer, and heated in a 100 °C water bath for 10 minutes. As for electrophoresis, we used the PE008 kit (Shaanxi Zhonghui Hecai Biomedical Technology Co., Ltd., China) to prepare the gel, and loaded the samples at a voltage of 80 V, then transferred the proteins using wet transfer system. After transferring, we washed the membrane with TBST and then blocked in 5% non-fat dry milk at room temperature for 1 hour. Then we added the primary antibody (anti-rabbit Bcl-2, ABclonal, A19693, 1:7000; anti-rabbit Bax, ABclonal, A19684, 1:2000; anti-rabbit cleaved caspase 3, Affinity, AF7022, 1:1000) and incubated at 4 °C for 6-8 hours. After washing the primary antibody with TBST, we added the corresponding secondary antibody (anti-rabbit IgG, HRP-linked Antibody, Cell Signaling Technology, 7074, 1:5000) and incubated at room temperature for 1 hour. After washing with TBST, we applied the ECL detection mixture onto the membrane and used the Servicebio (China) luminescence imaging workstation to detect the protein expression.

***Measurement of intracellular ATP levels*:** Intracellular ATP levels were measured using a commercial ATP detection kit (Beyotime, China) according to the manufacturer’s instructions. Briefly, cells were lysed on ice using ATP lysis buffer (200 μL per well of a 6-well plate). Cell lysates were collected and centrifuged at 12000 g for 5 min at 4 °C, and the supernatants were used for subsequent analysis. ATP standard solutions were prepared by serial dilution in ATP lysis buffer to generate a standard curve. The ATP detection working solution was freshly prepared by diluting the ATP detection reagent with the provided dilution buffer at a ratio of 1:9. For ATP measurement, 100 μL of ATP detection working solution was added to each well. Subsequently, 20 μL of sample or standard was added, mixed immediately, and luminescence was recorded using a luminometer (VICTOR Nivo, PerkinElmer, USA). ATP concentrations were calculated based on the standard curve. To normalize for variations in cell number or protein content, total protein concentration in each sample was determined using a BCA protein assay, and ATP levels were expressed as nmol ATP per mg protein. For relative comparison, the control group was normalized to 100%, and all other groups were expressed as percentages of the control.

***Mitochondrial membrane potential assay* *(JC-1)*:** Mitochondrial membrane potential was assessed using a JC-1 mitochondrial membrane potential assay kit (Beyotime, China). For ESC-RGCs with different treatments, culture medium was removed and cells were gently washed once with PBS when necessary. Cells were then incubated with 1 mL fresh culture medium followed by the addition of 1 mL JC-1 staining working solution and incubated at 37 °C for 20 min. During incubation, JC-1 staining buffer was prepared by diluting the 5× buffer with distilled water and kept on ice. After staining, cells were washed twice with JC-1 staining buffer and replenished with 2 mL culture medium. Fluorescence signals were observed using a fluorescence or confocal microscope. Because ESC-RGCs intrinsically express red fluorescent markers, only JC-1 monomer-associated green fluorescence was imaged and quantified to evaluate changes in mitochondrial membrane potential.

***Mitochondrial permeability transition pore (MPTP) assay*:** Opening of the mitochondrial permeability transition pore (MPTP) was evaluated using a calcein AM-based fluorescence assay kit (Beyotime, China). ESC-RGCs with different treatments were washed with PBS and incubated with calcein AM staining solution in the presence of a fluorescence-quenching reagent to selectively quench cytosolic calcein fluorescence, allowing retention of mitochondrial calcein signals. Cells were incubated at 37 °C in the dark for 30 min, followed by replacement with fresh prewarmed culture medium and an additional incubation at 37 °C for 30 min to ensure complete intracellular esterase-mediated hydrolysis of calcein AM. Cells were then washed with PBS and observed under a fluorescence microscope. A decrease in mitochondrial green fluorescence intensity indicated increased MPTP opening, whereas preserved calcein fluorescence reflected inhibition of MPTP opening and maintenance of mitochondrial integrity.

***RNA-Seq*:** The total RNA of PBS and Li-EGCG NPs groups of ESC-RGCs were extracted by Trizol (Takara Bio., Japan) according to the standard procedure. RNA-seq paired-end reads were sequenced on the Illumina NovaSeq X Plus PE150. Raw data (raw reads) of fastq format were firstly processed through fastp software, and subsequently mapped to the hg38 transcriptome using Hisat2 v2.0.5. Differentially expressed genes were identified using DESeq2, with significant genes defined by a *P* value < 0.05 and an absolute log2 fold change > 0. Data were scaled and visualized on the NovoMagic platform (https://magic-plus.novogene.com/). Gene ontology (GO) enrichment, Kyoto Encyclopedia of Genes and Genomes (KEGG), and Gene Set Enrichment Analysis (GSEA) analyses were conducted using clusterProfiler R package. Bubble chart, chord diagram and heatmap were plotted by https://www.bioinformatics.com.cn, an online platform for data analysis and visualization.

***Quantitative real-time PCR (qRT-PCR)*:** Cells were treated as indicated, and total RNA was isolated using Trizol (Takara Bio., Japan) according to the manufacturer’s protocol. Complementary DNA (cDNA) was synthesized using the RR037A cDNA Synthesis Kit (Takara Bio., Japan) in a PCR thermal cycler (37 °C for 15 min, followed by 85 °C for 5 s). The resulting cDNA was diluted and subjected to quantitative PCR using SYBR Green chemistry. Each reaction (10 μL total volume) contained 5 μL SYBR Green master mix, 0.5 μL each of forward and reverse primers, 1 μL cDNA template, and 3 μL nuclease-free water. qRT-PCR was performed on on a LightCycler® 480 Real-Time PCR System (Roche, USA). Relative mRNA expression levels were calculated using the 2^-ΔΔCT^ method, with *GAPDH* serving as the internal reference gene. Primer sequences are listed in **Table S1**.

**References**

[1] H. Liu, Y. Zhang, Y. Y. Zhang, Y. P. Li, Z. Q. Hua, C. J. Zhang, K. C. Wu, F. Yu, Y. Zhang, J. Su, Z. B. Jin,"Human embryonic stem cell-derived organoid retinoblastoma reveals a cancerous origin," *Proc Natl Acad Sci U S A* **2020**, *117* 33628-33638.

**2. Supplementary Table**

**Table S1.** Primers used in the qRT-PCR assay

| Gene | Organisms | Forward (5’-3’) | Reverse (5’-3’) |
| --- | --- | --- | --- |
| *GAPDH* | Homo sapiens | gtcaaggctgagaacgggaa | aaatgagccccagccttctc |
| *SOD2* | Homo sapiens | ggcctacgtgaacaacctga | ccgttagggctgaggtttgt |
| *GPX4* | Homo sapiens | cagtgaggcaagaccgaagt | ccgaactggttacacgggaa |
| *PRDX3* | Homo sapiens | GTTGTCGCAGTCTCAGTGGA | AACAGCACACCGTAGTCTCG |
| *NNT* | Homo sapiens | ACAGAAGACAGTGGCTGAGC | AATGCCAGCCAAGCCAAAAG |
| *BCL2L1* | Homo sapiens | GGTCGCATTGTGGCCTTTTT | GGTAAGTGGCCATCCAAGCT |
| *CXCL12* | Homo sapiens | CTACAGATGCCCATGCCGAT | CAGCCGGGCTACAATCTGAA |
| *CXCR4* | Homo sapiens | GCTGTTGGCTGAAAAGGTGG | ATCTGCCTCACTGACGTTGG |
| *MAPK3* | Homo sapiens | CTCTGCCCTCCAAGACCAAG | CCGTCGGGTCATAGTACTGC |
| *PAK4* | Homo sapiens | CTGCTCTTCAACGAGGTGGT | GGAACTCCATGACCACCCAG |
| *FZD9* | Homo sapiens | TTCTTCTCCACCGCCTTCAC | GAGAGGAAGATGATGGGGCG |

**3. Supplementary Figures**


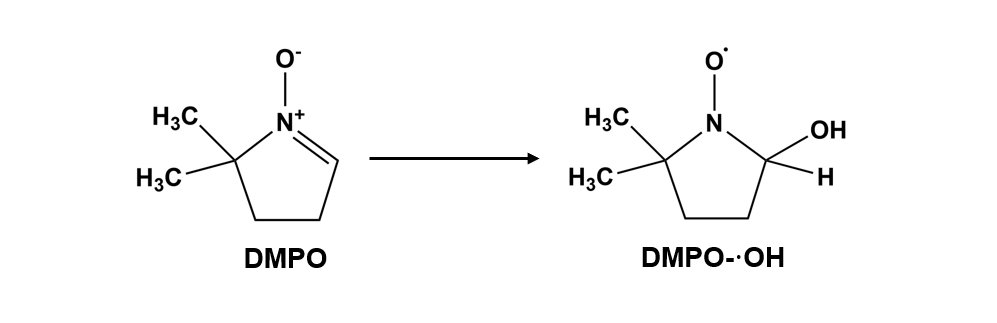


**Figure S1**. Chemical equations represent the redox reactions of ·OH in ESR experiment. ESR, electron spin resonance.


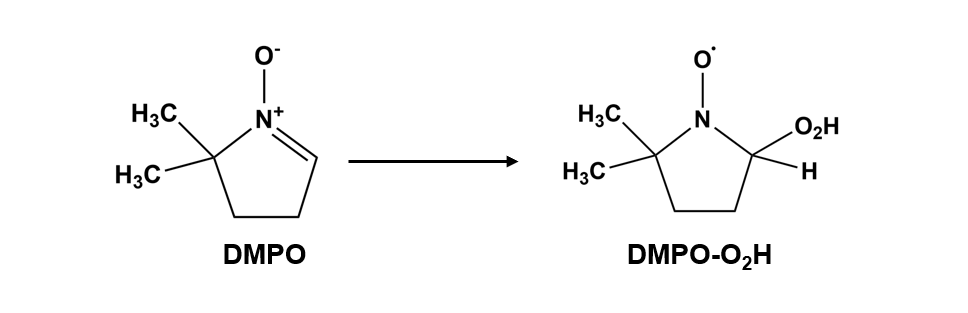


**Figure S2**. Chemical equations represent the redox reactions of ·O_2_- in ESR experiment. ESR, electron spin resonance.


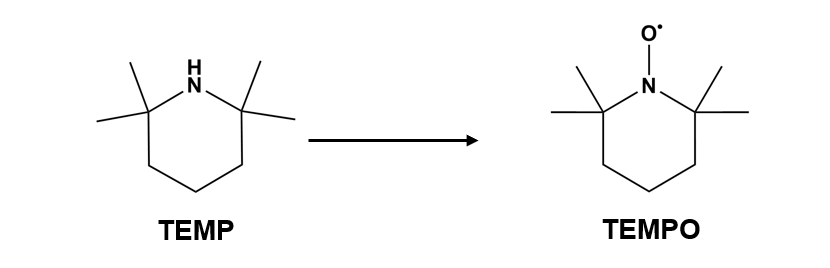


**Figure S3**. Chemical equations represent the redox reactions of ^1^O_2_ in ESR experiment. ESR, electron spin resonance.


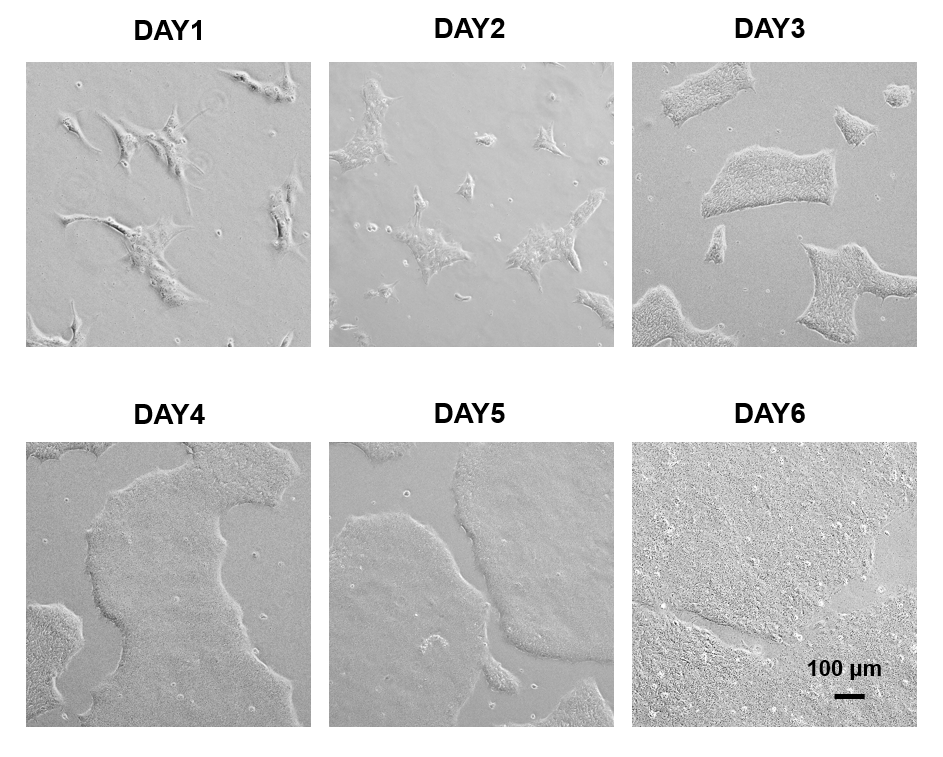


**Figure S4**. Colony-like morphology of ESCs (embryonic stem cells) under light microscopy, with densely packed cells and clear borders. Scale bar: 100 μm.


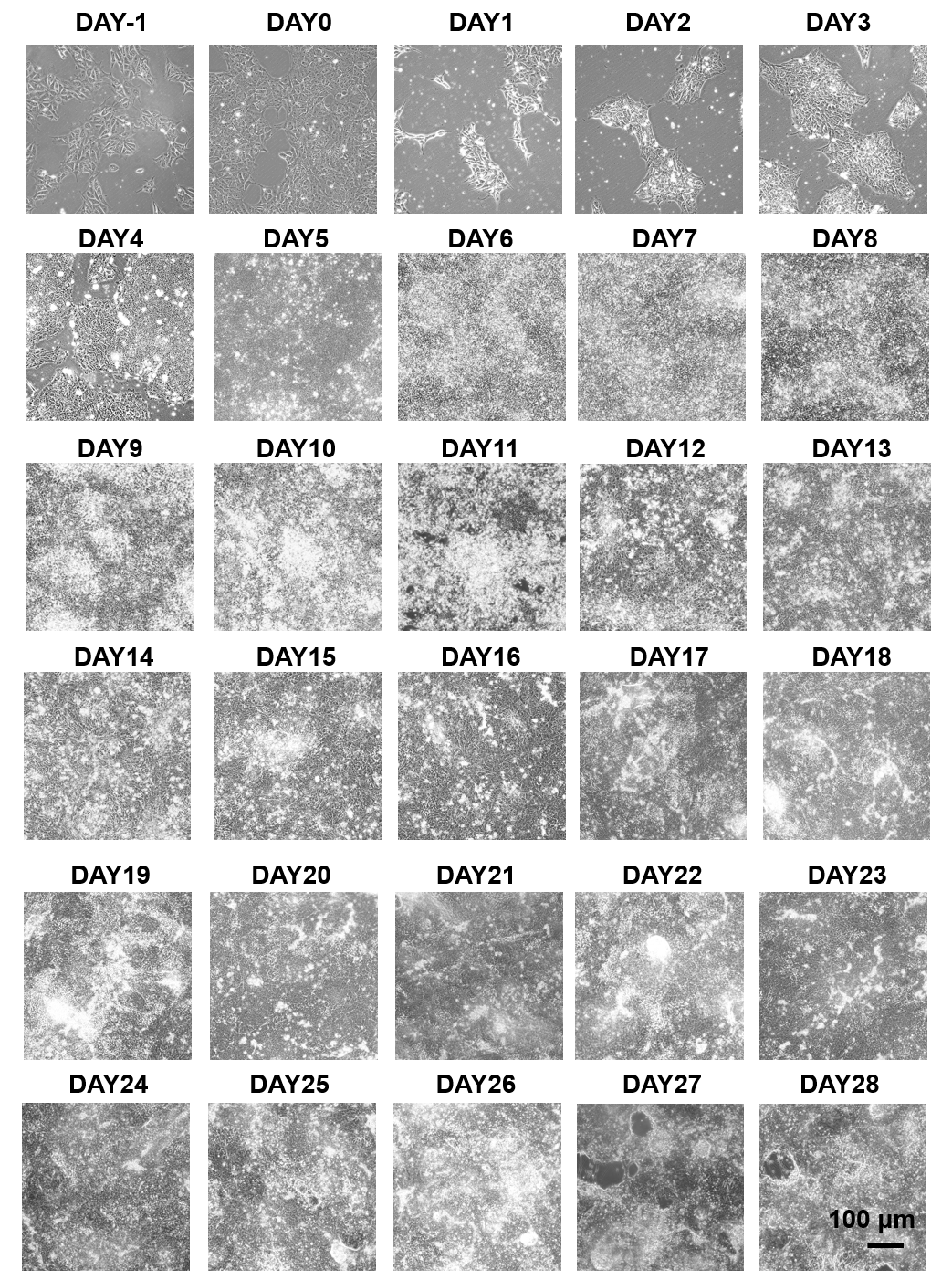


**Figure S5**. Representative images showing the morphological progression of ESC-RGCs from day -1 to day 28 during differentiation. Scale bar: 100 μm.


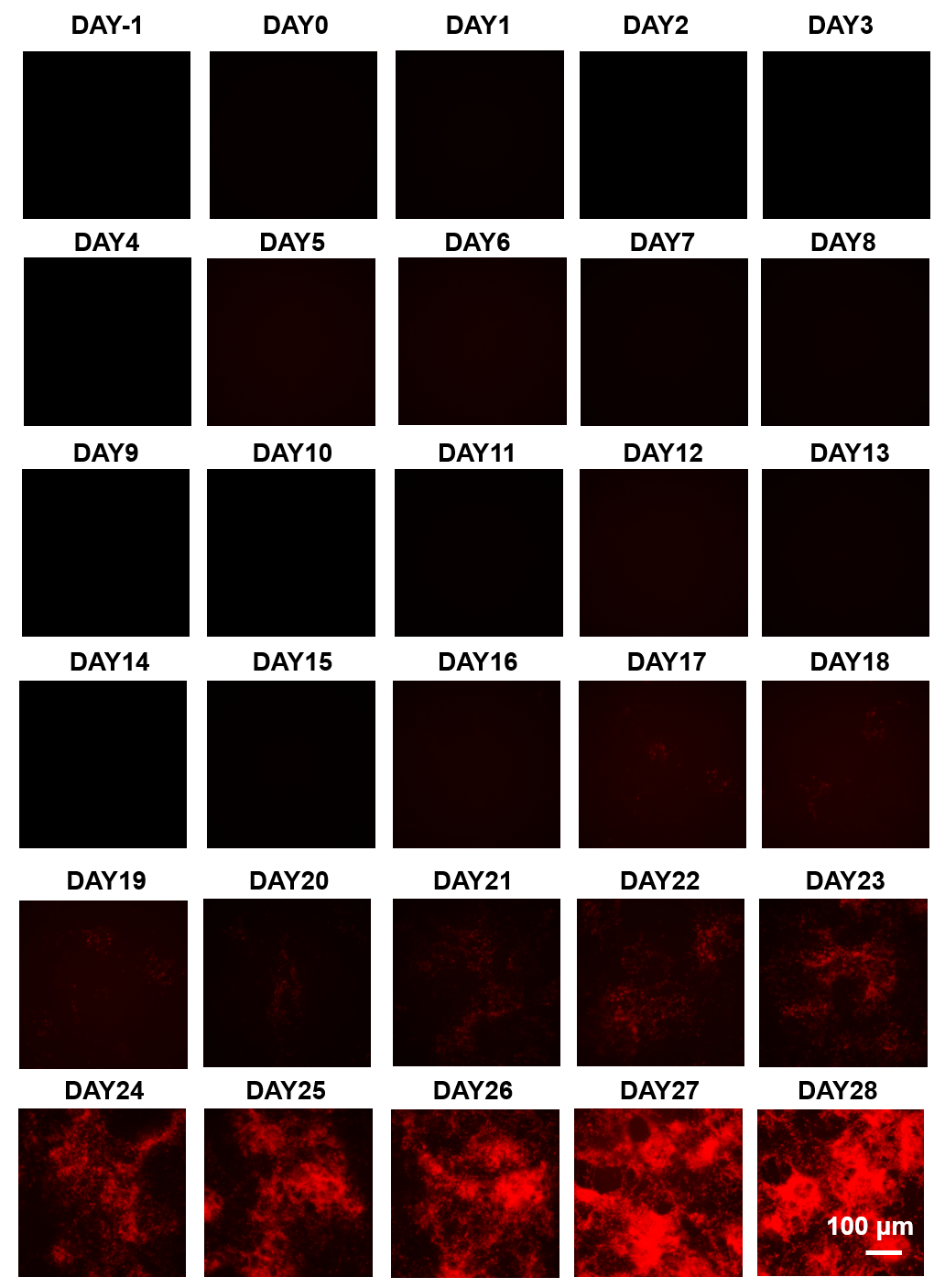


**Figure S6**. Red fluorescence images showing BRN3B-Tdtomato expression from day -1 to day 28. Scale bar: 100 μm.
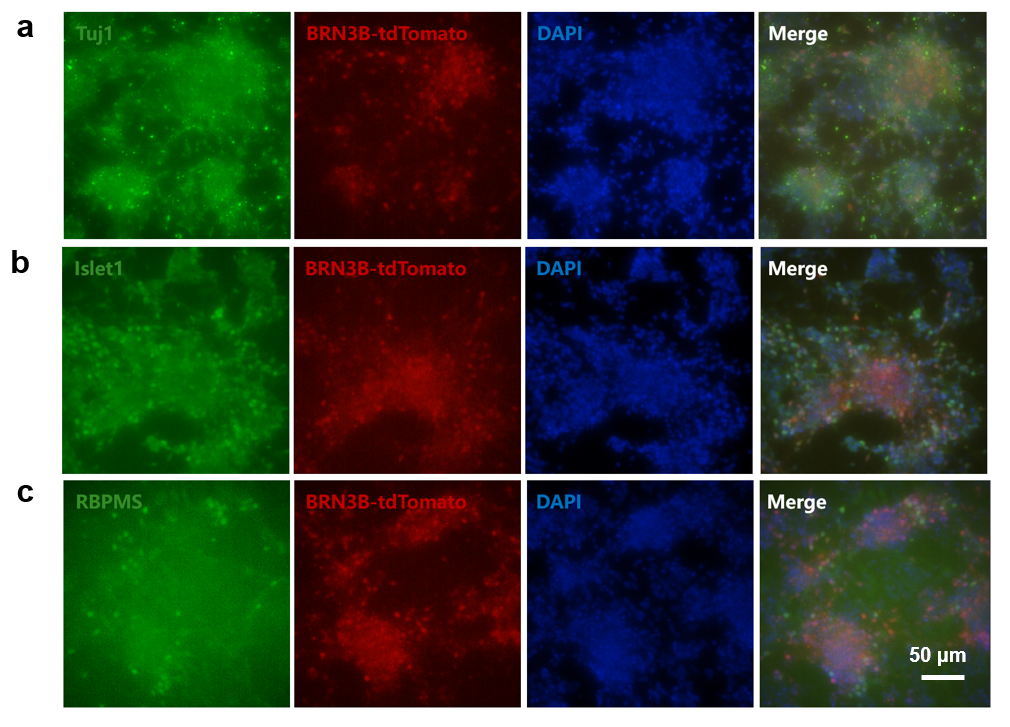


**Figure S7**. Identification of ESC-RGCs (embryonic stem cell derived retinal ganglion cells). a-c, Green indicates immunostaining for RGC markers Tuj1, Islet1, and RBPMS, respectively. Red represents BRN3B-tdTomato expression, and blue indicates DAPI nuclear staining. Scale bar: 50 μm.


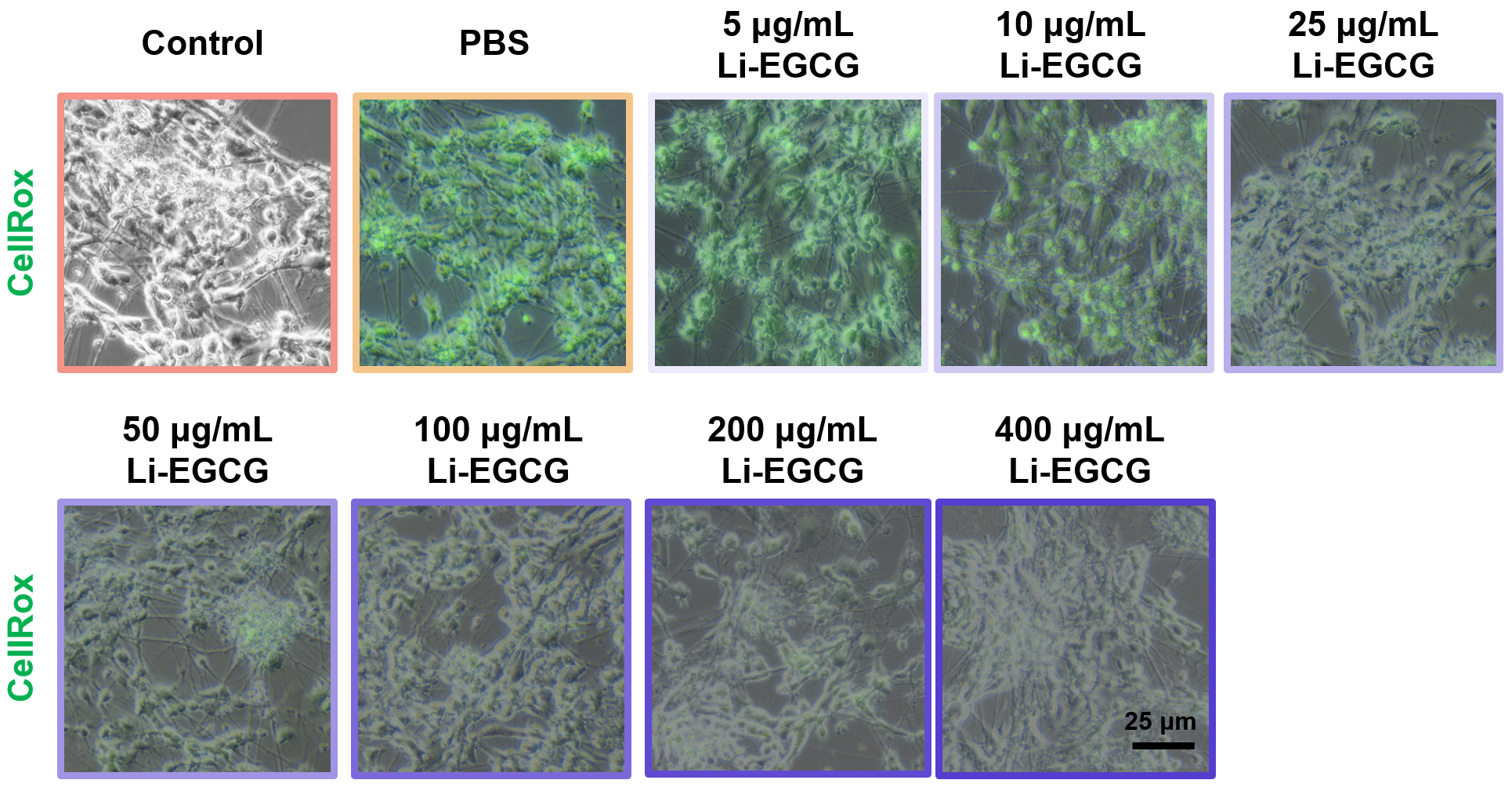


**Figure S8**. Representative images of oxidative stress markers CellRox (green) for overall cellular reactive ROS in ESC-RGCs of treatment with varying concentrations of Li-EGCG NPs.


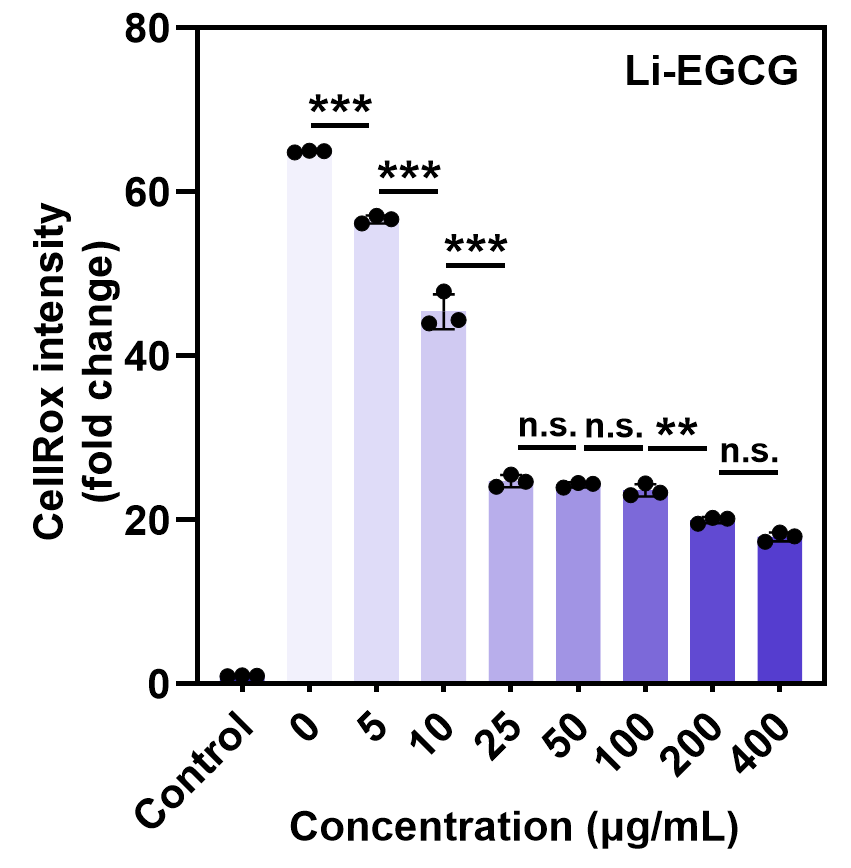


**Figure S9**. Quantitative analysis of ROS levels in ESC-RGCs of treatment with varying concentrations of Li-EGCG NPs based on fluorescence intensity of CellROX staining (*n* = 3). Data are presented as mean ± SD; statistical analysis was performed using one-way ANOVA followed by Tukey’s post hoc tests (**P* < 0.05, ***P* < 0.01, ****P* < 0.001, n.s. not significant).


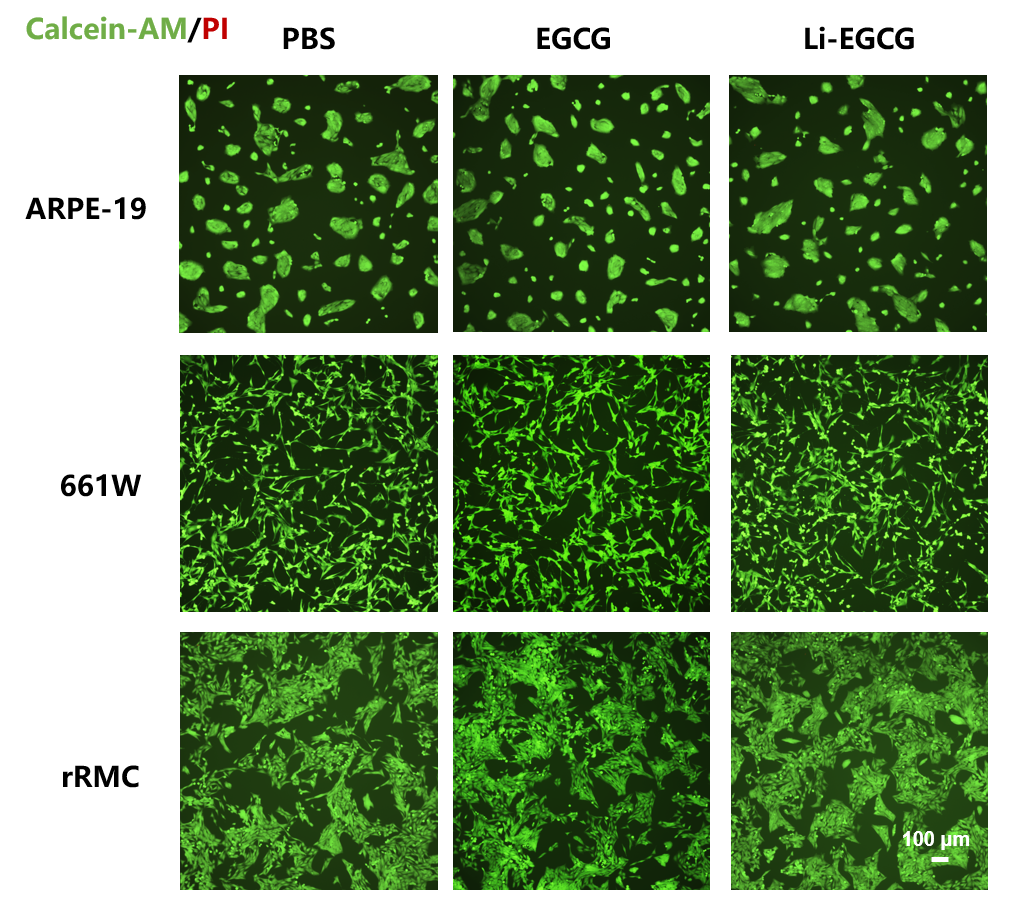


**Figure S10**. Safety evaluation of Li-EGCG NPs in ocular cell lines. Live/dead staining was performed in human retinal pigment epithelial cells (ARPE-19), mouse photoreceptor cells (661W), and rat Müller glial cells (rRMC). Calcein acetoxymethyl ester/propidium iodide (Calcein-AM, green) labels live cells, and propidium iodide (PI, red) labels dead cells. The results show high cell viability in all ocular cell lines after Li-EGCG treatment. Scale bar: 100 μm.


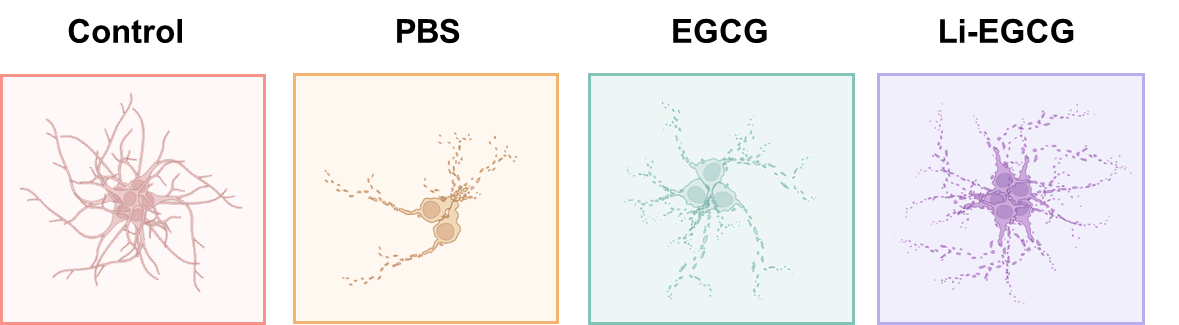


**Figure S11**. Schematic illustration of neurite morphological changes across treatment groups.


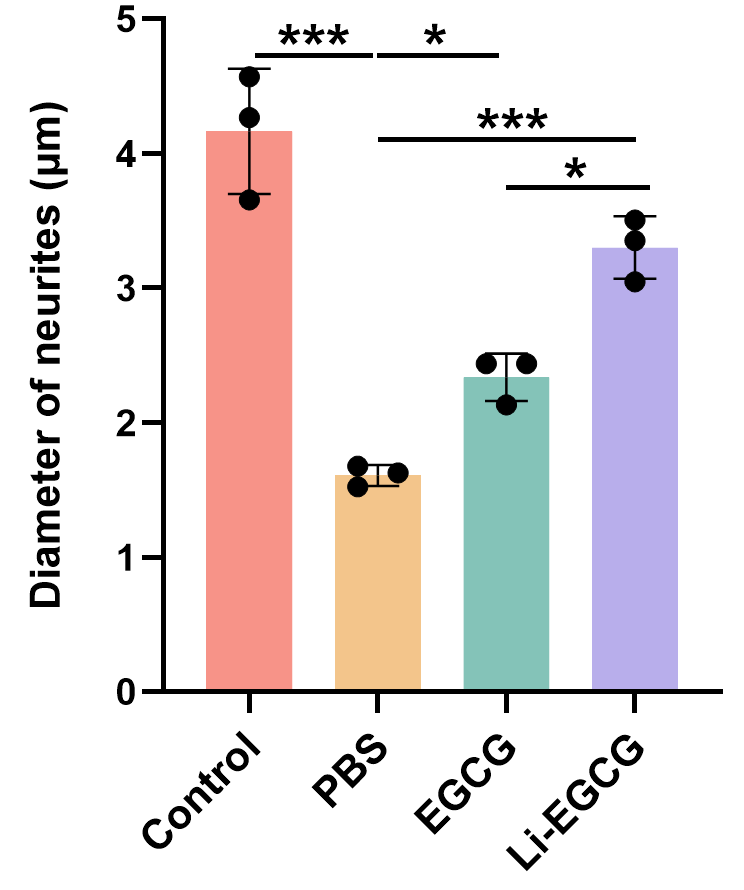


**Figure S12**. Quantification of neurite diameter in ESC-RGCs subjected to different treatments (*n* = 3). Data are presented as mean ± SEM. Statistical significance was determined using one-way ANOVA followed by Tukey’s post hoc tests (*n* = 3, **P* < 0.05, ***P* < 0.01, ****P* < 0.001).


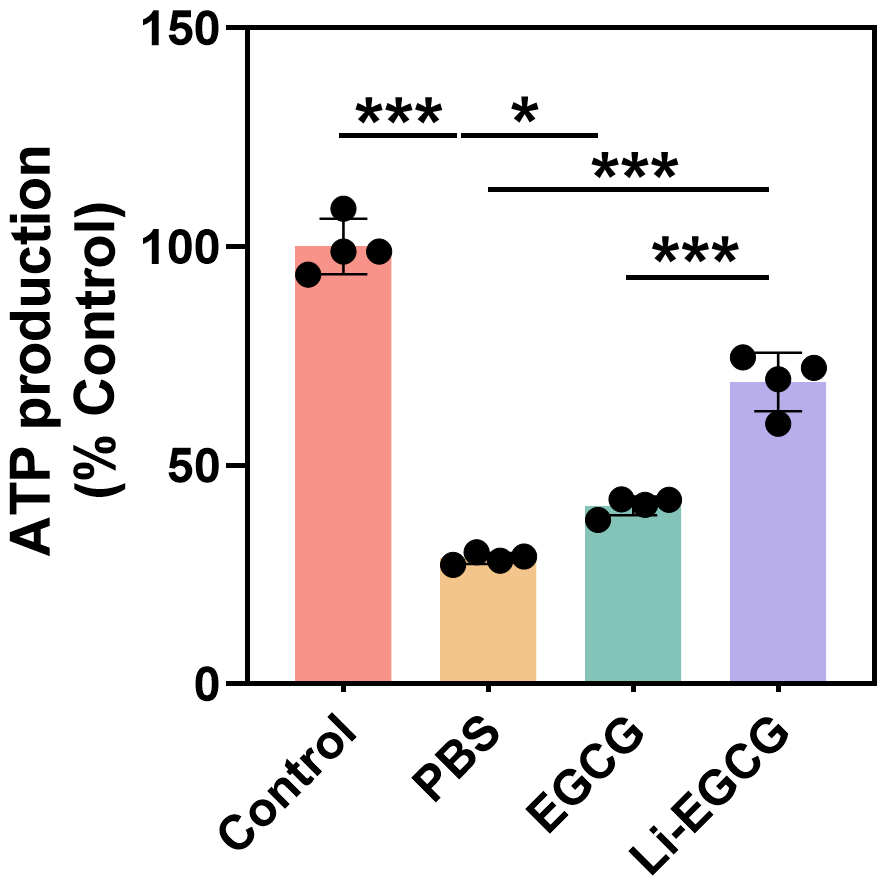


**Figure S13**. Quantitative analysis of intracellular ATP production in each group, expressed as a percentage relative to the control group (*n* = 4). Data are presented as mean ± SEM. Statistical significance was determined using one-way ANOVA followed by Tukey’s post hoc tests (*n* = 3, **P* < 0.05, ***P* < 0.01, ****P* < 0.001).


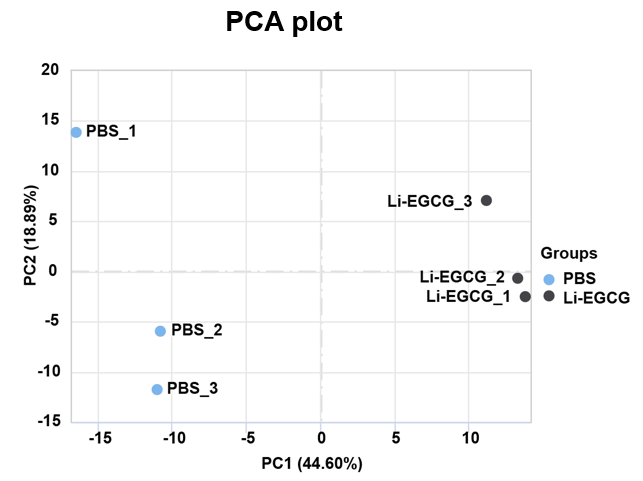


**Figure S14**. Principal component analysis (PCA) plot shows clear clustering of samples, indicating good reproducibility.


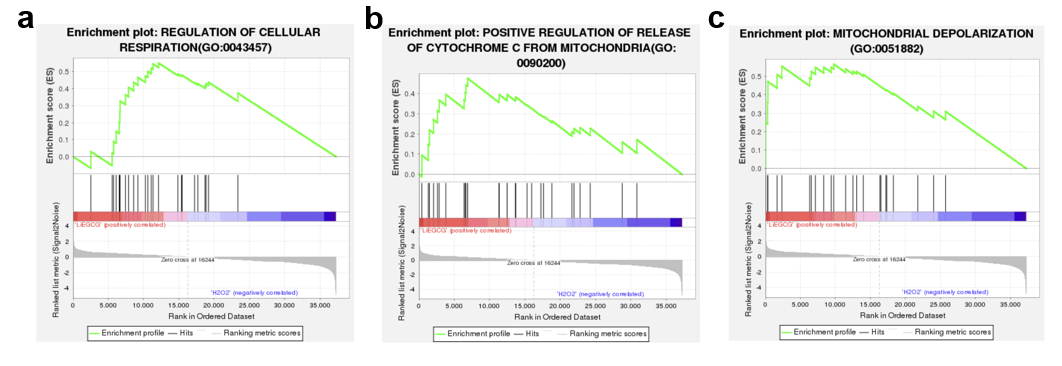


**Figure S15**. Gene Set Enrichment Analysis (GSEA) of up regulated genes in the Li-EGCG NPs group. The results show significant enrichment in Gene Ontology (GO) terms related to (a) cellular respiration, (b) mitochondrial cytochrome c release, and (c) mitochondrial depolarization. All enrichments are statistically significant with *P* < 0.01.


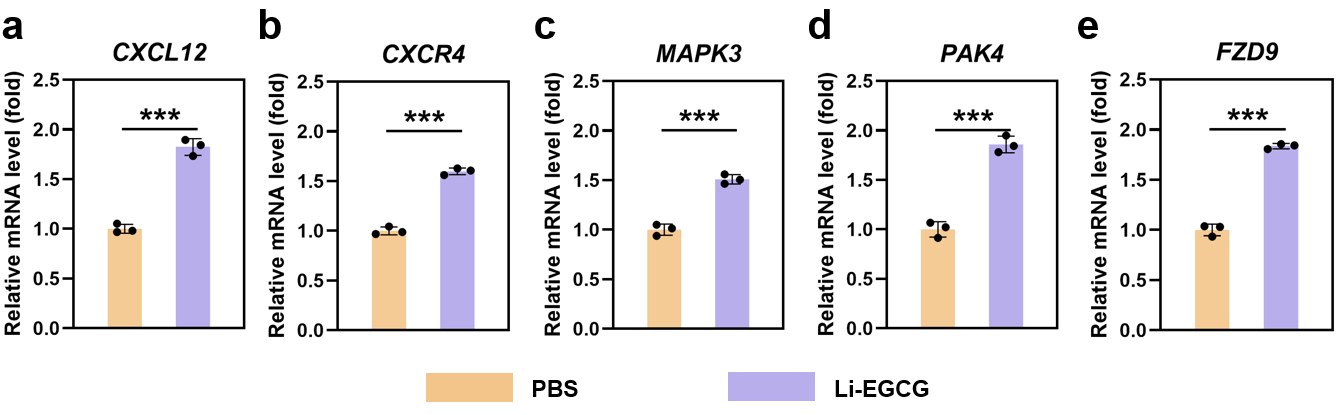


**Figure S16**. Quantitative analysis of the mRNA expression of *CXCL12* (a), *CXCR4* (b), *MAPK3* (c), *PAK4* (d), and *FZD9* (e) in PBS and Li-EGCG groups (*n* = 3). Data are presented as mean ± SD; statistical analysis was performed using Student’s t-tests (**P* < 0.05, ***P* < 0.01, ****P* < 0.001).


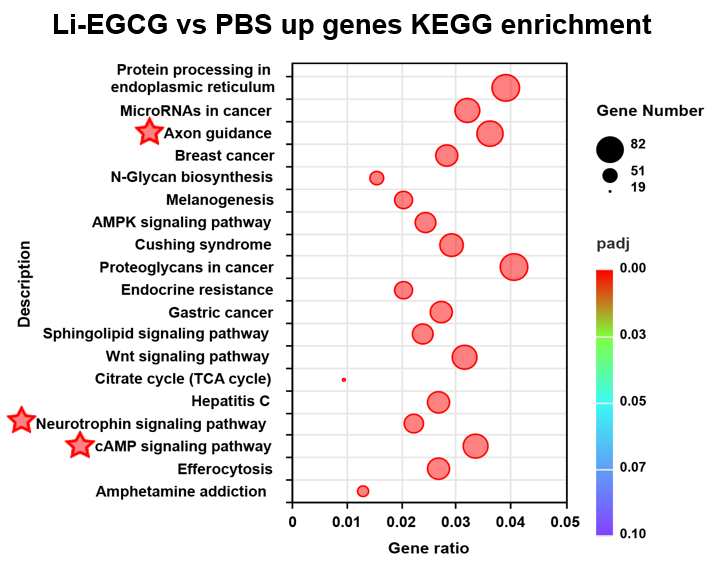


**Figure S17**. Kyoto Encyclopedia of Genes and Genomes (KEGG) pathway enrichment analysis shows increased activity in pathways such as axon guidance, neurotrophin signaling pathway, and cAMP signaling pathway.


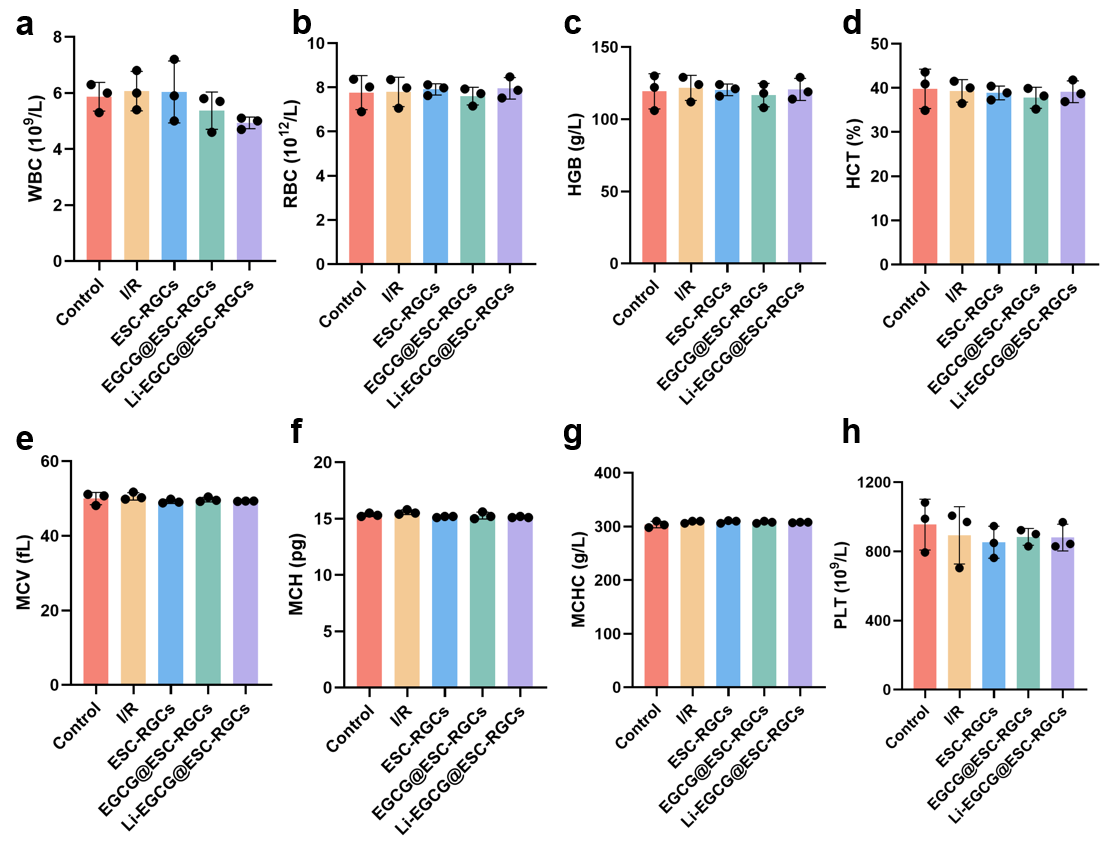


**Figure S18**. a-h) Complete blood count results for Control, I/R, ESC-RGCs, EGCG@ESC-RGCs, and Li-EGCG@ESC-RGCs groups (*n* = 3). No significant differences were observed among groups for the measured parameters, as determined by one-way ANOVA.


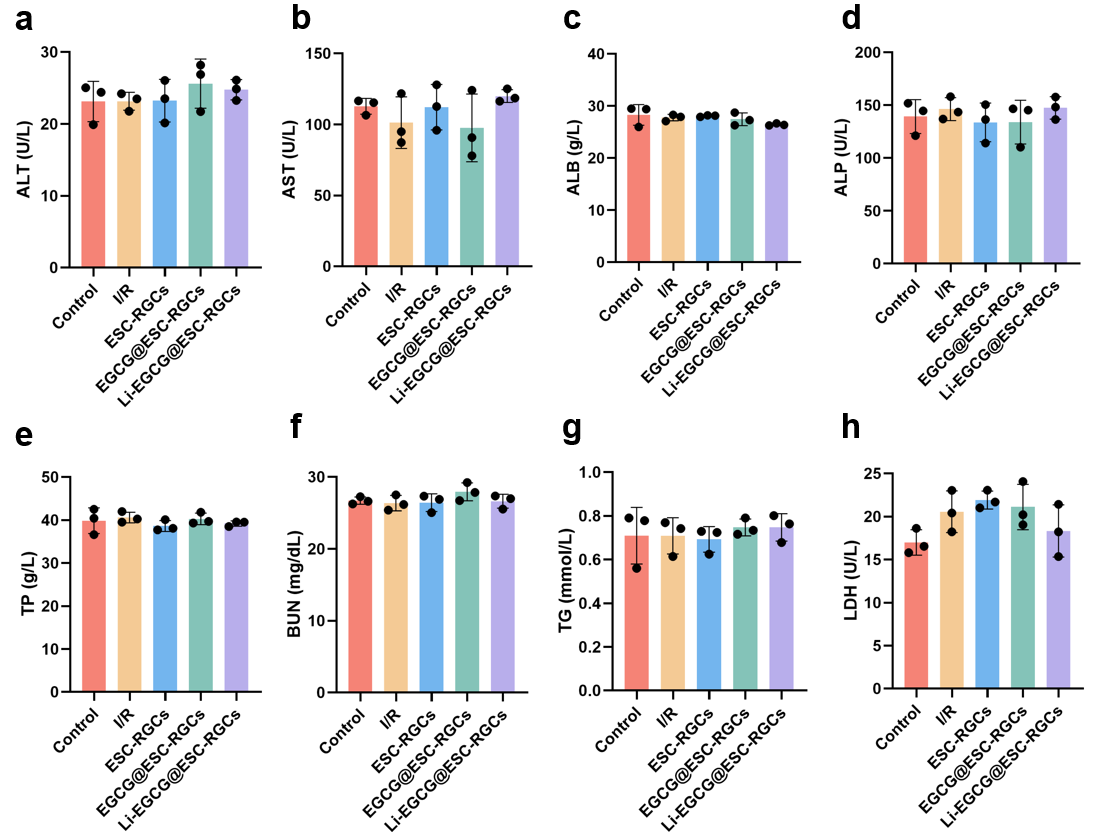


**Figure S19**. a-h) Serum biochemistry results for Control, I/R, ESC-RGCs, EGCG@ESC-RGCs, and Li-EGCG@ESC-RGCs groups (*n* = 3). No significant differences were observed among groups for the measured parameters, as determined by one-way ANOVA.


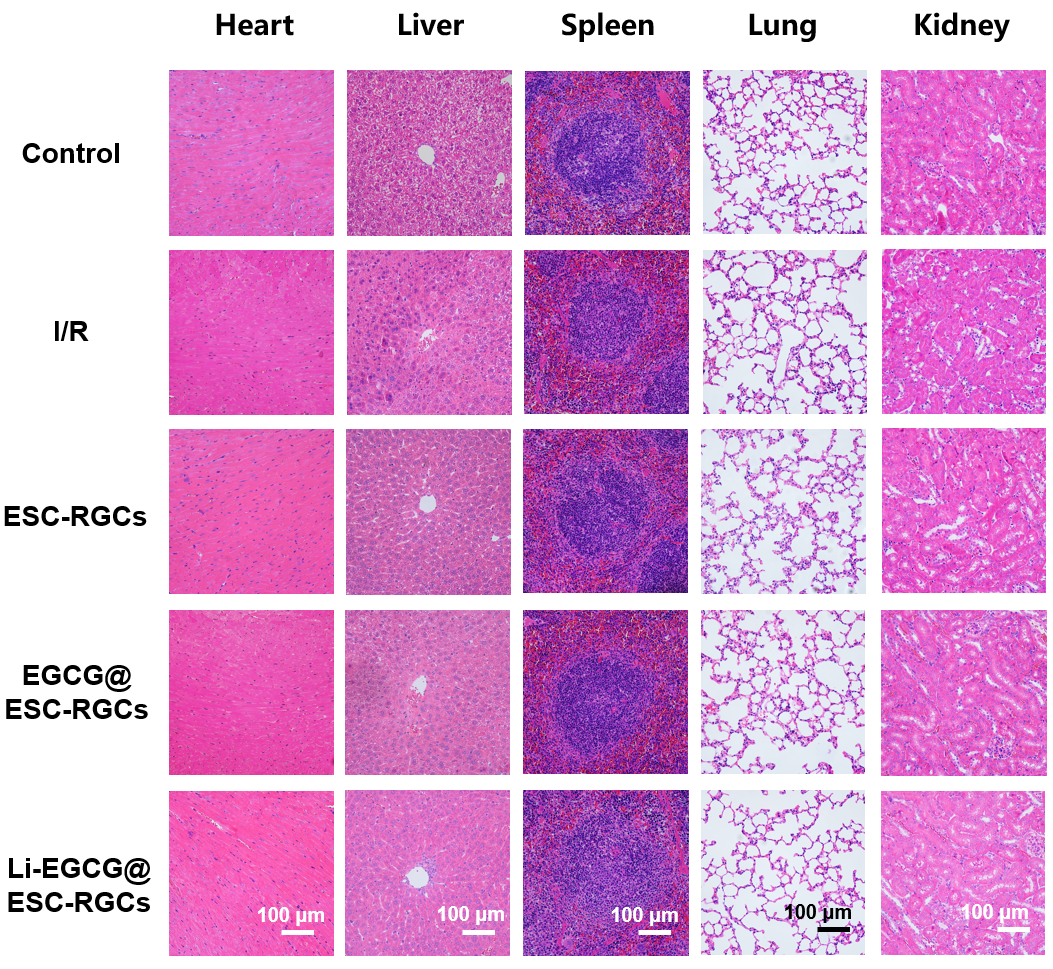


**Figure S20**. In vivo biocompatibility of Li-EGCG@ESC-RGCs injection. Representative images of the H&E staining in the heart, liver, spleen, lung and kidney of the Control, I/R, ESC-RGCs, EGCG@ESC-RGCs, Li-EGCG@ESC-RGCs groups. Scale bars: 100 μm.


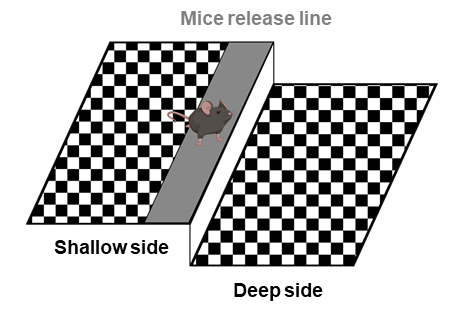


**Figure S21**. Schematic of the visual cliff test: mice were released from the center line and the time spent on the deep side was recorded.


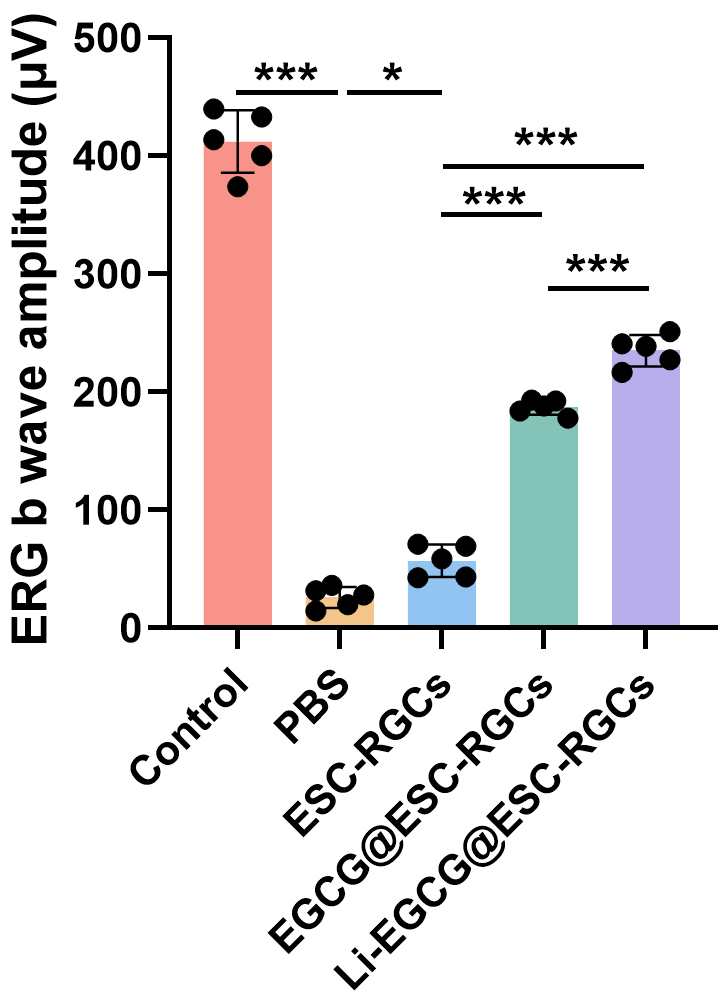


**Figure S22**. Quantitative analysis of scotopic ERG b-wave amplitudes (*n* = 5). Data are presented as mean ± SD; statistical analysis was performed using one-way ANOVA followed by Tukey’s post hoc tests (**P* < 0.05, ***P* < 0.01, ****P* < 0.001).
